# Evolution of spin excitations from bulk to monolayer FeSe

**DOI:** 10.1038/s41467-021-23317-3

**Published:** 2021-05-25

**Authors:** Jonathan Pelliciari, Seher Karakuzu, Qi Song, Riccardo Arpaia, Abhishek Nag, Matteo Rossi, Jiemin Li, Tianlun Yu, Xiaoyang Chen, Rui Peng, Mirian García-Fernández, Andrew C. Walters, Qisi Wang, Jun Zhao, Giacomo Ghiringhelli, Donglai Feng, Thomas A. Maier, Ke-Jin Zhou, Steven Johnston, Riccardo Comin

**Affiliations:** 1 Department of Physics, Massachusetts Institute of Technology, Cambridge, MA USA; 2 NSLS-II, Brookhaven National Laboratory, Upton, NY USA; 3 Center for Nanophase Materials Sciences, Oak Ridge National Laboratory, Oak Ridge, Tennessee USA; 4 State Key laboratory of Surface Physics and Department of Physics, Fudan University, Shanghai, China; 5 Dipartimento di Fisica, Politecnico di Milano, Milano, Italy; 6 Quantum Device Physics Laboratory, Department of Microtechnology and Nanoscience, Chalmers University of Technology, Göteborg, Sweden; 7 Diamond Light Source, Harwell Campus, Didcot, UK; 8 CNR-SPIN, Dipartimento di Fisica, Politecnico di Milano, Milano, Italy; 9 Computational Sciences and Engineering Division, Oak Ridge National Laboratory, Oak Ridge, TN USA; 10 Department of Physics and Astronomy, The University of Tennessee, Knoxville, TN USA

**Keywords:** Electronic properties and materials, Superconducting properties and materials, Surfaces, interfaces and thin films

## Abstract

In ultrathin films of FeSe grown on SrTiO_3_ (FeSe/STO), the superconducting transition temperature *T*_c_ is increased by almost an order of magnitude, raising questions on the pairing mechanism. As in other superconductors, antiferromagnetic spin fluctuations have been proposed to mediate SC making it essential to study the evolution of the spin dynamics of FeSe from the bulk to the ultrathin limit. Here, we investigate the spin excitations in bulk and monolayer FeSe/STO using resonant inelastic x-ray scattering (RIXS) and quantum Monte Carlo (QMC) calculations. Despite the absence of long-range magnetic order, bulk FeSe displays dispersive magnetic excitations reminiscent of other Fe-pnictides. Conversely, the spin excitations in FeSe/STO are gapped, dispersionless, and significantly hardened relative to its bulk counterpart. By comparing our RIXS results with simulations of a bilayer Hubbard model, we connect the evolution of the spin excitations to the Fermiology of the two systems revealing a remarkable reconfiguration of spin excitations in FeSe/STO, essential to understand the role of spin fluctuations in the pairing mechanism.

## Abstract (web summary)

Here, Pelliciari et al. present resonant inelastic X-ray scattering on monolayer samples of unconventional superconductor FeSe, finding evidence for gapped and dispersionless spin excitations. These experiments are very difficult due to the extremely small scattering volume of the FeSe monolayer.

## Introduction

**Fig. 1:**
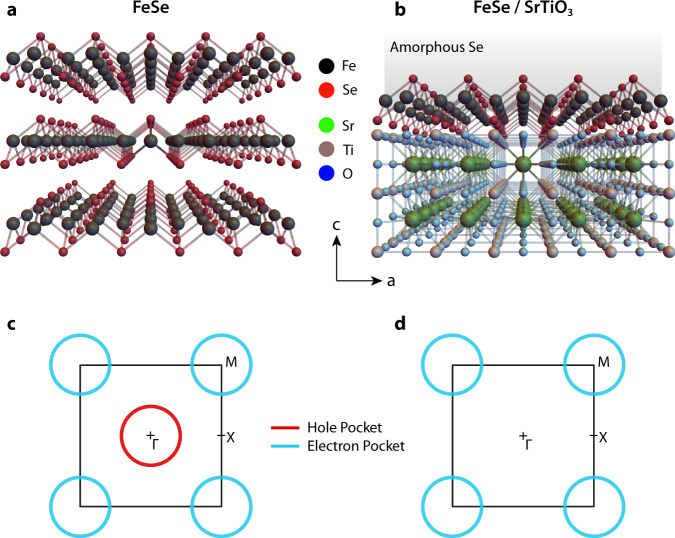
Structure and Fermi surface of FeSe bulk and FeSe/SrTiO_3_ (STO). **a** Structure of FeSe bulk. **b** Structure of FeSe/STO monolayer with Se capping. **c**, **d** Schematic Fermi surface of FeSe bulk (**c**) and FeSe/STO monolayer (**d**). The electron pocket of bulk FeSe has been drawn circular and not elliptical for simplicity and for correspondence with the theoretical model adopted here.

Iron selenide (FeSe) occupies a unique place among Fe-based superconductors. It has the simplest structure, consisting of a square Fe lattice with Se ions situated above and below it, as depicted in Fig. 1a. It is superconducting with *T*_c_ ~ 8 K and has a structural transition at *T*_s_ ~ 90 K^1–3^. The Fermi surface of bulk FeSe is composed of cylindrical hole pockets at the Γ point and elliptical electron pockets at the *M* point (see Fig. 1c; hereafter, a Brillouin zone with two Fe sites per unit cell is adopted). The Fermi surface of FeSe/STO, on the other hand, is composed solely of circular electron pockets at the *M *point^4–9^, while the hole pockets at the Γ point are pushed below the Fermi level (Fig. 1d). These observations are consistent with an electron doping of ~ 0.1/Fe, as extracted from the Luttinger count^4,5,9^, suggesting that STO acts as an electron donor for monolayer FeSe.

Simultaneous Néel- and stripe-like fluctuations have been observed in bulk FeSe at **q** = (1, 0) and (1, 1) (reciprocal lattice units, r.l.u.), despite the lack of long-range antiferromagnetic order. These observations signal the presence of significant magnetic frustration that ultimately precludes any long-range order^3^. From an experimental perspective, the investigation of spin excitations in FeSe/STO is complicated by the limited volume contributing to the magnetic scattering signal. Inelastic neutron scattering (INS) is currently unable to probe single atomic layers, and other light scattering techniques, such as Raman and optical spectroscopy, cannot disentangle the signals from the substrate, the FeSe layer, and the interface between the two. On this front, recent advances in Resonant Inelastic X-ray Scattering (RIXS) have allowed the detection of spin excitations in Fe-based superconductors, producing complementary information to INS^10–18^. The signal enhancement and sensitivity to electronic excitations that is afforded by resonant photoexcitation render RIXS a prime technique for investigating ultrathin materials. Additionally, the elemental selectivity of RIXS enables one to isolate the signal from specific atoms and disentangle the contributions from the film and the substrate. These aspects make RIXS an ideal technique for studying magnetic excitations in FeSe/STO.

Here, we combine high-energy-resolution RIXS measurements and quantum Monte Carlo (QMC) calculations within the dynamical cluster approximation (DCA) to elucidate the spin dynamics of bulk FeSe and FeSe/STO films down to the single unit cell limit. We find that the magnetic excitations in FeSe/STO are gapped and dispersionless in momentum space, and harden significantly relative to other Fe-based superconductors. These observations are in stark contrast with the spin excitations of bulk FeSe, which exhibit an acoustic-like dispersion toward the zone center, similarly to other antiferromagnetic systems^1^. The evolution of the spin excitations is captured by DCA calculations of a bilayer Hubbard model^19^, which account for the transition from a two-band system into an incipient band system (see Methods). Correspondingly, we establish that the reconfiguration of the spin excitations from bulk to monolayer FeSe originates from the Lifshitz transition of the Fermi surface and accompanying loss of the hole pocket at the Γ point. This transition quenches particle-hole scattering processes, flattens and gaps out their dispersion, and increases their energy bandwidth, in agreement with the experimental observations.

## Results

### X-Ray absorption spectroscopy

**Fig. 2:**
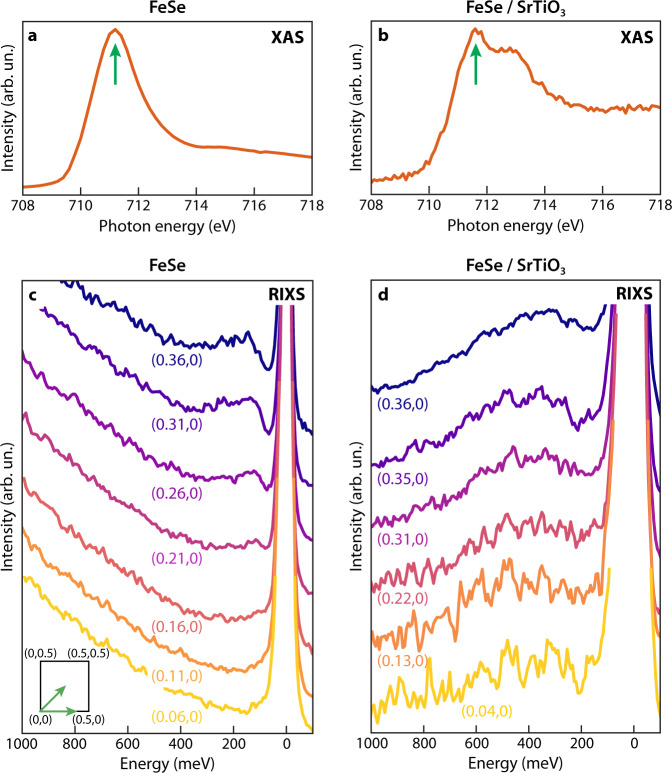
X-Ray Absorption Spectroscopy (XAS) and Resonant Inelastic X-Ray Scattering (RIXS) spectra for FeSe bulk and Fe/SrTiO_3_ (STO). **a**, **b** Fe *L*_3_-edge X-ray absorption spectra for FeSe bulk (**a**) and FeSe/STO (**b**), measured via total electron yield. The arrows mark the incident energy for the RIXS data displayed in **c** and **d**. **c**, **d** High-energy resolution RIXS spectra of FeSe bulk (**c**) and FeSe/STO (**d**) at different momentum points along the high-symmetry direction (0, 0) → (*H*, 0) [RIXS spectra along the (0, 0) → (*H*, *H*) direction are reported in the Supplementary Information].

Figure 2a, b summarize the Fe *L*-edge X-ray Absorption Spectroscopy (XAS) data for bulk FeSe (FeSe hereafter) and monolayer FeSe (FeSe/STO hereafter), respectively. The XAS of FeSe resembles the spectra previously obtained from cleaved Fe pnictides crystals with Fe in a 2+ oxidation state and embedded in a metal environment^10,11,13,15,16^. The XAS of FeSe/STO has an additional peak at higher energy, which could originate from new interfacial valence states induced by hybridization with orbitals of the STO substrate. The arrows in Fig. 2a, b specify the incident photon energies at which RIXS spectra were collected.

### Resonant Inelastic X-Ray Scattering

Figure 2c, d show the corresponding high-resolution and high-statistics RIXS data on FeSe and monolayer FeSe/STO, respectively. In the bulk case, we detect a dispersive excitation at an energy of ~140 meV at **q** = (0.36, 0) r.l.u., which gradually decreases in energy toward the zone center until it merges into the elastic line. This mode is reminiscent of what observed in INS experiments^3^ and can be ascribed to spin excitations as previously shown in ref. ^14^. A word of caution should be given, however, as FeSe lacks long-range antiferromagnetism, and, instead exhibits Néel- and stripe-type fluctuations^3^. As such, a direct comparison between the excitations measured by INS and RIXS is not straightforward since the Γ point is not equivalent to *M* or *X* in the absence of Brillouin zone folding. Nevertheless, the excitations of FeSe closely resemble those observed in BaFe_2_As_2_^14^, suggesting that spin fluctuations are of similar nature in these two compounds in proximity of the Γ point and across the portion of Brillouin zone accessible to RIXS.

**Fig. 3:**
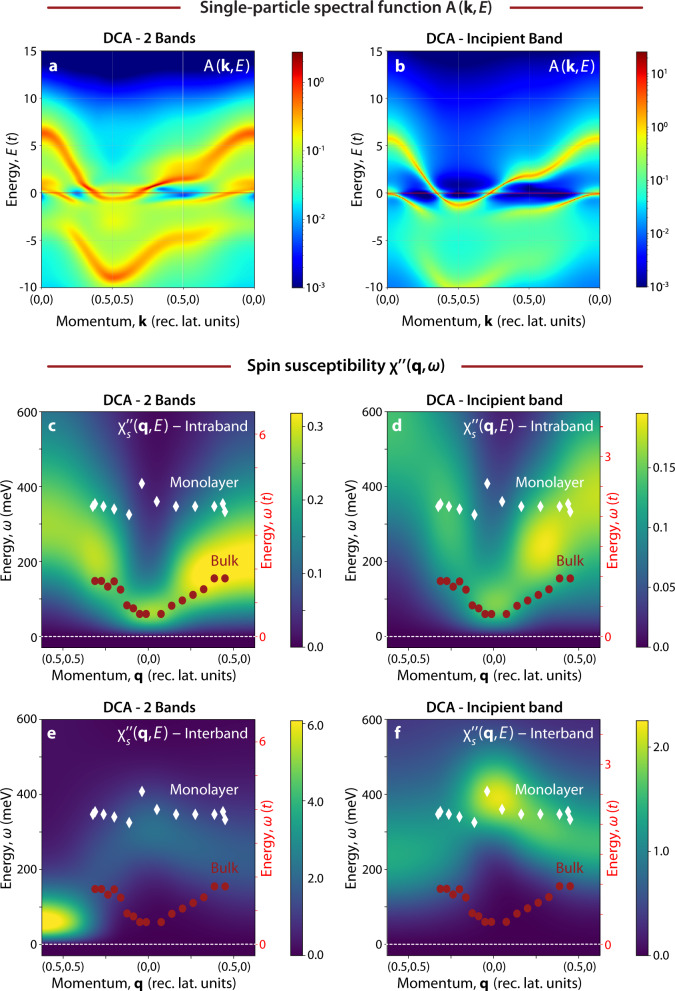
Single-particle spectral function and dynamical spin susceptibility from Dynamical Cluster Approximation (DCA) calculations. **a**, **b** DCA calculations and spectral function *A*(**k**, *E*) for the two-band Hubbard model (**a**) and the incipient band Hubbard model (**b**). **c**–**f** DCA calculations of the imaginary part of the spin susceptibility ${\chi }_{s}^{\prime\prime} ({\bf{q}},\omega )$ for the two-band Hubbard model (**c**: intraband *Q*_*z*_ = 0; **e** interband *Q*_*z*_ = *π*) and the incipient band Hubbard model (**d** intraband *Q*_*z*_ = 0; **f** interband *Q*_*z*_ = *π*). Red circles (white diamonds) indicate the energy position of the peak detected by Resonant Inelastic X-Ray Scattering (RIXS) in bulk (monolayer) FeSe. The uncertainties associated with peak fitting are smaller than the markers.

We observe significant differences in the RIXS spectra collected on the FeSe monolayer. At zero energy loss, we detect a strong elastic signal that likely reflects the overall diffuse scattering from the capping layer, the FeSe film, and the STO substrate. Despite this strong elastic background, we are able to identify inelastic peaks owing to the high energy resolution of the instrument (~40 meV). In particular, we observe a broad peak located at ~320 meV at **q** = (0.36, 0) r.l.u., whose energy linewidth is significantly greater than the excitations detected in the bulk case. This peak is largely asymmetric – similar to bulk FeSe – but its tail extends to energies as high as 1 eV, much higher than the bulk counterpart. Furthermore, this mode barely disperses as a function of momentum and has an energy of ~320−400 meV along the (*H*, 0) and (*H*, *H*) directions, as reported in Figs. 2d and 3. Thanks to resonant photoexcitation at the Fe-*L* edge, we can identify the FeSe layer as the host of this excitation. This interpretation is further supported by the dependence of the RIXS signal on the incident photon energy across the resonance (see Supp. Inf.). The ability to make this assignment is essential to disentangle excitations originating from the film, the substrate or the interface.

The evolution of the spin excitations from FeSe bulk to monolayer is significant and cannot be compared nor ascribed to any doping effects previously observed in related materials. For example, the spin excitations of BaFe_2_As_2_ evolve differently depending on the doping type: in the case of hole doping (K-), the spin excitations gradually soften upon doping^10,20^, electron doping (Co/Ni-) leaves the high-energy spin excitations more or less unaffected^20–22^ while in the isovalent doped case (P-) the spin excitations harden gradually^15,23^. Nonetheless, the doping-induced changes observed in these systems are minor compared to the effect observed here. The hardening of spin excitations in P-doped BaFe_2_As_2_ (40 meV) – so far the largest reported in the literature – is much smaller than what we observe in FeSe. Most importantly, a clear dispersion is found in these compounds at all doping levels, contrary to the flat momentum dependence in the FeSe monolayer.

### Qunatum Monte Carlo calculations

The principal difference between FeSe and FeSe/STO is in their band structure and Fermi surface topology. To explore the impact of these differences on the spin excitations, we calculated the single-particle spectral function *A*(**k**, *E*) and dynamical spin susceptibility ${\chi }_{s}^{^{\prime\prime} }({\bf{q}},\omega )$ of the bilayer Hubbard model using the dynamical cluster approximation (DCA) and a nonperturbative QMC solver (see Methods). The bilayer Hubbard model is the simplest model with an electronic structure similar to the Fe-based superconductors that can be studied with QMC while maintaining a manageable sign problem. By varying the value of the nearest-neighbour interlayer hopping *t*_⊥_, the electronic structure of the model can be tuned from a system with both hole- and electron-like bands crossing the Fermi level (Fig. 3a) to one with a single electron-like band crossing the Fermi level and an incipient hole band (Fig. 3b). The model can, therefore, capture the qualitative features of the band structure of bulk and monolayer FeSe. In Fig. 3a, we report the spectral function for the two-band model, where we observe a hole-like band crossing the Fermi level close to the Γ point and an electron-like band intersecting the Fermi level in proximity of the *M* point. This band structure leads to a double pocket Fermi surface as sketched in Fig. 1c. In the case of the incipient band model, shown in Fig. 3b, the hole band at the Γ point is pushed to lower energies, moving below the Fermi level and removing the hole pocket at the Γ point. The resulting Fermi surface is composed only of a circular electron pocket at the *M* point, as sketched in Fig. 1d.

**Fig. 4:**
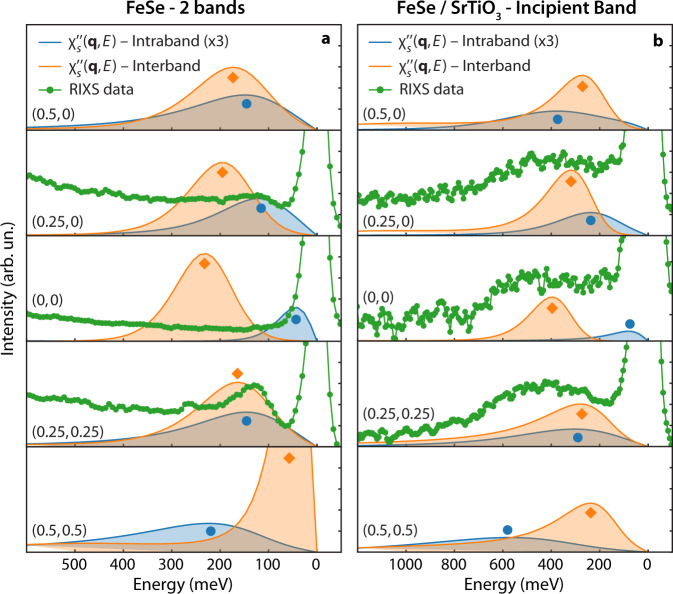
Comparison of spin susceptibility and Resonant Inelastic X-Ray Scattering (RIXS) data. **a** Dynamical Cluster Approximation (DCA) calculations of the imaginary part of the spin susceptibility ${\chi }_{s}^{\prime\prime} ({\bf{q}},\omega )$ for the incipient band Hubbard model (blue lines: intraband *k*_*z*_ = 0; orange lines: interband *k*_*z*_ = *π*) and **b** the two-band Hubbard model (blue lines: intraband *k*_*z*_ = 0; orange lines: interband *k*_*z*_ = *π*). Orange (blue) circles indicate the energy position of the maximum of the spin susceptibility in the interband (intraband) cases.

Figures 3c–f and 4 display the calculated imaginary part of the spin susceptibility ${\chi }_{s}^{\prime\prime} ({\bf{q}},\omega )$ spectra for two values of *t*_⊥_, corresponding to bulk and FeSe/STO. In our model, two components of ${\chi }_{s}^{\prime\prime} ({\bf{q}},\omega )$ are extracted with intra- (*q*_*z*_ = 0) and interband (*q*_*z*_ = *π*) character, which can be isolated from one another by choosing the appropriate value of *q*_*z*_. Figure 3c–f report the intra- and interband channels in the middle and bottom rows, respectively. In the case of the two-band model with two ambipolar Fermi pockets, we obtain a strongly dispersing ${\chi }_{s}^{\prime\prime} ({\bf{q}},\omega )$ (see Figs. 3c, e and 4b), whose main two components—arising from intraband and interband scattering—disperse out-of-phase in momentum space. Specifically, the intraband component has a minimum at the Γ point and increases in energy towards its maximum at (0.5, 0) and (0.5, 0.5) while the interband component displays two minima at (0.5, 0) and (0.5, 0.5) and a maximum at (0, 0). An analysis of the spectral intensity reveals that the interband component is four to five times larger than the intraband one. This difference is highlighted in the line cuts plot reported in Fig. 4b, where both the intra- and interband components are displayed and the former has been multiplied by a factor three for better visualization.

Upon increasing *t*_⊥_, the hole-like band is made incipient. The interband component of the resulting ${\chi }_{s}^{\prime\prime} ({\bf{q}},\omega )$ is much less dispersive and becomes gapped throughout the entire Brillouin zone, in close agreement with the experimental findings (see Figs. 3f and 4a). The out-of-phase dispersion of the intra- and interband ${\chi }_{s}^{\prime\prime} ({\bf{q}},\omega )$ is also preserved for the incipient band condition. The difference of the dispersion relation of the two susceptibilities (inter- and intra-) is larger than those observed by changing the system from a two-band model to an incipient band one. The large difference between the intra- and interband spin susceptibility is preserved even for small variations of the model parameters, as shown in the Supp. Inf. This behavior can be rationalized once one recognizes that the a channel for particle-hole scattering will be closed once the hole-like band sinks below the Fermi level. Finally, we note that the width of the spectra of our ${\chi }_{s}^{\prime\prime} ({\bf{q}},\omega )$ captures the broadening of the peaks in the incipient band case compared to the two-band model.

Figure 3c–f summarize our results by comparing the calculated inter- and intra-band ${\chi }_{s}^{\prime\prime} ({\bf{q}},\omega )$ as a false color image, with experimental peak positions overlaid. Here, the results are shown for both bulk (white circles) and monolayer (white diamonds) FeSe. (A more detailed description of the extraction of the experimental data points is given in the Supp. Inf.) We have assumed *t* = 90 (160) meV for the bulk (incipient) case when converting the DCA energy scale to physical units, which produces the best agreement with the experimental data. The use of different factors for the two cases is supported by recent DMFT+LDA calculations, which indicate that bulk FeSe is more correlated than the FeSe/STO^24^. This conclusion is also consistent with our observation of much sharper spectral functions in the incipient band case, see Fig. 3a, b. Consequently, it is natural to adopt a larger *t* for the monolayer case while holding the value of *U* fixed. We note that our choice of *t* = 90 (160) meV corresponds to Hubbard repulsion values of *U* = 8*t* = 0.72 (1.28) eV. This value is significantly renormalized down from the average value $\bar{U}=3{-}4$ eV obtained in first-principles calculations for the Fe-based superconductors^25–27^; however, our model value is comparable to the value $\bar{U}\approx 0.5{-}0.6$ obtained when the same calculations are downfolded onto a space containing only the Fe 3*d* orbitals^25^ by integrating out the highly polarizable O 2*p* and As/Se 4*p* orbitals. Our value is also consistent with those needed to reproduce the experimental value of *T*_*c*_ in a recent FLEX study of the bilayer Hubbard model^28^. We, therefore, conclude that our energy scales are consistent with effective model treatments of the Fe-based superconductors.

### Comparison of experimental data and calculations

In Fig. 4 we show a comparison of the spin susceptibility calculations with the experimental data plotted as a line cut. The experimental dispersion in bulk FeSe appears to be in better agreement with the intraband ${\chi }_{s}^{\prime\prime} ({\bf{q}},\omega )$ (Fig. 3c, d and blue traces in Fig. 4b) rather than the interband component (Fig. 3e, f and orange traces in Fig. 4b). The intensity of the interband ${\chi }_{s}^{\prime\prime} ({\bf{q}},\omega )$ is higher than the intraband ${\chi }_{s}^{\prime\prime} ({\bf{q}},\omega )$ and one might expect that the RIXS signal scales proportionally. However, matrix elements of the RIXS cross-section have not been included in the model, which makes a qualitative comparison the only viable option. Including Fe-L edge matrix elements would require a momentum-resolved full multi-orbital Fe calculation, which is currently not possible due to the severe Fermion sign problem induced by Hund’s coupling. To better understand the behaviour of the proposed models and how the RIXS intensity is related to the intra- versus inter-band susceptibility, we studied a simplified ladder model using exact diagonalization. The ladder model can be viewed as a 1D analog to the bilayer model considered here. We compute the RIXS spectra for these ladders using the Kramers-Heisenberg formula and a relatively large cluster to obtain reasonable momentum resolution. The results (summarized in the Supp. Inf.) establish a clear connection between the RIXS intensity and the dynamical structure factor and support the use of the spin susceptibility when examining our multi-orbital model. Moreover, these simplified ladder calculations confirm that transitioning from a two-band to an incipient band model suppresses the intraband susceptibility. When taken together, these results indicate that the 2D bilayer Hubbard model can be used to describe our experimental results qualitatively.

In any case, from a phenomenological perspective, the agreement of the experimental data with the intraband ${\chi }_{s}^{\prime\prime} ({\bf{q}},\omega )$ from a bilayer Hubbard model is good and future calculations including orbital orientation and polarization effects could offer a more quantitative description of the RIXS cross-section. In Figs. 3d, f and 4a, we report the calculations obtained for the incipient band model (tailored for FeSe/STO), where the agreement between theory and experiments is better for the interband ${\chi }_{s}^{\prime\prime} ({\bf{q}},\omega )$ (orange lines in Fig. 4a). In this case, the interband ${\chi }_{s}^{\prime\prime} ({\bf{q}},\omega )$ is flattened by the lack of the hole pocket and the hardening of the dispersion is reproduced by the theory. In Fig. 4a we also use circles to mark the two components of the spin susceptibility, which highlight the agreement between the RIXS data of Fe/STO with the interband susceptibility. These changes are a direct consequence of the fact that intraband scattering is strongly suppressed at low-energies once the hole pocket is shifted below the Fermi level. This hardening and flattening of the electronic excitations is clearly observed in the experimental data for FeSe/STO as corroborated by the white diamonds overlaid with the color plot (Fig. 3). The interband ${\chi }_{s}^{\prime\prime} ({\bf{q}},\omega )$ also has the largest intensity compared to the intraband ${\chi }_{s}^{\prime\prime }({\bf{q}},\omega )$, and is, therefore, expected to dominate the RIXS signal when neglecting cross-section effects.

## Discussion

Our findings have implications for the enhancement of SC in FeSe/STO. In Eliashberg- and fluctuation exchange-type models (FLEX), ${\chi }_{s}^{\prime\prime} ({\bf{q}},\omega )$ enters directly into the equation to calculate *T*_c_^19,29^. The significant evolution in ${\chi }_{s}^{\prime\prime} ({\bf{q}},\omega )$ revealed by our RIXS data suggests a sizable change this input of the equation, highlighting the importance of spin excitations for a complete explanation and description of SC in FeSe/STO. Moreover, any quantitative model for the spin fluctuation contribution to pairing must also account for the observed evolution of the spin dynamics. As such, the evolution of the spin dynamics from FeSe to FeSe/STO represents an essential clue to a magnetic-like pairing scenario, which was previously proposed for other Fe pnictides^1,29–34^. The present results do not, however, rule out additional interactions such as phonons or doping from the substrate, which can contribute to the enhancement of *T*_c_^35,36^.

In summary, we report a combined experimental and theoretical investigation of the spin dynamics in bulk FeSe and single-unit-cell FeSe/STO, uncovering a dramatic evolution of magnetic excitations from the bulk to the monolayer limit. In bulk FeSe, we observed dispersive spin excitations that are reminiscent of other Fe-based superconductors. These modes become significantly more energetic and less dispersive in the ultrathin limit of the FeSe/STO film. Quantum Monte Carlo calculations of the bilayer Hubbard model reveal that this reconfiguration of spin dynamics is a direct consequence of the modifications to interband scattering once the hole pocket is removed from the Fermi level. These findings suggest a fundamental link between the Fermiology of FeSe superconductors and their spin dynamics up to a very high energy scale. The direct experimental insights of the present RIXS study underscore the role of spin excitations for unconventional SC in FeSe, and provide an empirical benchmark for theoretical models of SC in FeSe/STO.

## Methods

### Sample preparation

#### Monolayer FeSe on STO

Monolayer of FeSe was grown on Nb-doped (0.5 wt%) (001)-oriented SrTiO_3_ substrate. The substrate was etched following the method described in ref. ^37^. In the growth chamber, which has a base pressure of 6 × 10^−10^ mbar, the substrate was heated to 800 ^∘^C for 45 min with Se flux. Single-layer FeSe films were grown at ~ 500 ^∘^C by coevaporation of Se and Fe with a flux ratio of 20: 1. After growth, the films were annealed at 550 ^∘^C in vacuum for 2 h. The FeSe/STO was characterized by ARPES and the superconducting gap was determined to be ~13.4 meV or *T*_c_ ~ 60−65 K. A ~25 nm thick layer of amorphous Se was added for protection at room temperature.

#### FeSe bulk

Bulk FeSe single crystals were grown under a permanent gradient of temperature (~400−330 ^∘^C) in the KCl–AlCl_3_ flux, as reported in ref. ^3^. The *T*_c_ of the bulk FeSe sample is ~8 K.

#### High energy resolution RIXS measurements on FeSe bulk and FeSe/STO

High-resolution RIXS experiments were performed at the I21-RIXS beamline at Diamond Light Source, United Kingdom. FeSe bulk was cleaved in vacuum. All samples were aligned with the surface normal (001) lying in the scattering plane. X-ray absorption was measured using the total electron yield (TEY) method by recording the drain current from the samples. For RIXS measurements, *π* polarized light was used. The combined energy resolution was about 40 meV (FWHM) at the Fe *L*_3_ edge (~710.5 eV). To enhance the RIXS throughput, a parabolic mirror has been installed in the main vacuum chamber. The RIXS spectrometer was positioned at a fixed scattering angle of 154 degrees resulting in a maximal total momentum transfer value **Q** of ~0.7 Å^−1^. The projection of the momentum transfer, **q**, in the *ab* plane was obtained by varying the incident angle on the sample. We use the 2 Fe unit cell convention with *a* = *b* = 3.76 Å and *c* = 5.4 Å for the reciprocal space mapping. The momentum transfer **Q** is defined in reciprocal lattice units (r.l.u.) as **Q** = **H**a^*^ + **K**b^*^ + **L**c^*^ where a^*^ = 2*π*/*a*, b^*^ = 2*π*/*b*, and c^*^ = 2*π*/*c*. All measurements were performed at 20 K under a vacuum pressure of about 5 × 10^−10^ mbar.

Spectra for the FeSe have been acquired in ~30 min whereas spectra for the FeSe/STO required 3 h or more for every momentum point.

#### Calculations

We modeled the spin excitation spectrum of bulk and monolayer FeSe using a two-orbital Hubbard model defined on a two-dimensional square lattice with *N* = *L*^2^ unit cells, where *L* is the linear size of the system. This model includes only the intraorbital Hubbard repulsion *U* on each orbital, and it is identical to the one used in ref. ^19^. (Details are also provided in the Supp. Inf. for completeness). Due to the orbital symmetry of the Hamiltonian, and the restriction to only a local intra-orbital Hubbard interaction, one can regard this model as a bilayer Hubbard model with layers *α* = 1, 2^19^. The kinetic energy term can then be diagonalized and rewritten in terms of a bonding *k*_*z*_ = 0 and anti-bonding *k*_*z*_ = *π* basis. As such, momentum transfers with *q*_*z*_ = 0 and *π* correspond to intra- and interband excitations, respectively. Throughout, we use *t* = 1 as the unit of energy, set *U* = 8*t*, and vary *t*_⊥_ and the filling *n* to control the electronic structure of the system.

We simulated the model using the dynamical cluster approximation (DCA) method^38^, where the bulk lattice system is mapped onto a periodic finite-size cluster embedded in a mean-field. The effective cluster problem was solved self-consistently by means of a continuous-time auxiliary field (CTAUX) quantum Monte Carlo method^39–41^. The real frequency dynamical correlation functions shown here were obtained from QMC data using the Maximum Entropy (MaxEnt) method^42^.

In our model we extracted the spectral function and dynamical susceptibilities for two different parameter sets $\frac{{U}}{{t}}=6$ and 8. Both parameter sets produced a two-band and an incipient band model in agreement with ARPES results. The susceptibility displayed very similar trends in terms of dispersion with modifications concerning the extent of the dispersion. This corroborates that these models are robust against reasonable variations of the parameters.

We additionally performed calculations using a 1D Hubbard ladder to confirm the RIXS sensitivity to the spin susceptibility and the suppression of intraband susceptibility in the incipient band model.

## Supplementary information

Supplementary Information

## Data Availability

Data that support the findings of this study are available upon reasonable request from the corresponding authors.

## Code availability

Codes that support the findings of this study are available upon reasonable request from the corresponding authors.
