# Supplementary material for: Evolution of spin excitations from bulk to monolayer FeSe
Source: Nat Commun. 2021 May 25;12:3122. doi: 10.1038/s41467-021-23317-3 (PMC8149670; doi:10.1038/s41467-021-23317-3)
Supplement: Supplementary file 1 — Supplementary Information [file 41467_2021_23317_MOESM1_ESM.pdf]

Supplementary Information for "Evolution of spin excitations from bulk to monolayer FeSe"

Jonathan Pelliciari,[1,2,*] Seher Karakuzu,[3] Qi Song,[4] Riccardo Arpaia,[5,6] Abhishek Nag,[7] Matteo Rossi,[5] Jiemin Li,[7] Tianlun Yu,[4] Xiaoyang Chen,[4] Rui Peng,[4] Mirian García-Fernández,[7] Andrew C. Walters,[7] Qisi Wang,[4] Jun Zhao,[4] Giacomo Ghiringhelli,[5,8] Donglai Feng,[4] Thomas A. Maier,[9,3] Ke-Jin Zhou,[7] Steven Johnston,[10] and Riccardo Comin[1,†]

[1] *Department of Physics, Massachusetts Institute of Technology, Cambridge, MA 02139, USA*

[2] *NSLS-II, Brookhaven National Laboratory, Upton, NY 11973, USA*

[3] *Center for Nanophase Materials Sciences, Oak Ridge National Laboratory, Oak Ridge, Tennessee 37831-6164, USA*

[4] *State Key laboratory of Surface Physics and Department of Physics, Fudan University, Shanghai, 200433, China*

[5] *Dipartimento di Fisica, Politecnico di Milano, I-20133 Milano, Italy*

[6] *Quantum Device Physics Laboratory, Department of Microtechnology and Nanoscience, Chalmers University of Technology, SE-41296 Göteborg, Sweden*

[7] *Diamond Light Source, Harwell Campus, Didcot OX11 0DE, United Kingdom*

[8] *CNR-SPIN, Dipartimento di Fisica, Politecnico di Milano, I-20133 Milano, Italy*

[9] *Computational Sciences and Engineering Division, Oak Ridge National Laboratory, Oak Ridge, Tennessee 37831-6164, USA*

[10] *Department of Physics and Astronomy, The University of Tennessee, Knoxville, TN 37996, USA*

I. BILAYER HUBBARD MODEL CALCULATIONS

We modeled the spin excitation spectrum of bulk FeSe and monolayer FeSe (FeSe/STO) using a two-orbital Hubbard model defined on a two-dimensional square lattice with $N = L^2$ unit cells, where L is the linear size of the system. The Hamiltonian is given by

$$\mathcal{H} = \mathcal{H}_{\mathcal{K}} + \mathcal{H}_{\mathcal{U}}, \tag{1}$$

where

$$\mathcal{H}_{\mathcal{K}} = t \sum_{\substack{\langle i,j\rangle \\ \sigma,\alpha}} \left(c^{\dagger}_{i,\alpha,\sigma} c_{j,\alpha,\sigma} + \text{H.c.} \right) + t_{\perp} \sum_{i,\sigma} \left(c^{\dagger}_{i,1,\sigma} c_{i,2,\sigma} + \text{H.c.} \right) - \mu \sum_{i\sigma,\alpha} n_{i,\alpha,\sigma} \,, \text{ and}$$

$$\mathcal{H}_{\mathcal{U}} = U \sum_{i,\alpha} n_{i,\alpha,\uparrow} n_{i,\alpha,\downarrow} \,.$$

Here, $c^{\dagger}_{i,\alpha,\sigma}$ ($c_{i,\alpha,\sigma}$) creates (annihilates) a spin σ electron in the unit cell i and orbital α, t is the intra-orbital hopping integral, $\langle i, j\rangle$ indicates a sum over nearest-neighbors, $t_{\perp}$ is the inter-orbital hopping integral between the orbitals on the same site, $n_{i,\alpha,\sigma} = c^{\dagger}_{i,\alpha,\sigma} c_{i,\alpha,\sigma}$ is the number operator, and U is the Hubbard interaction, which acts only between electrons in the same orbital. The total particle number is controlled by the chemical potential term μ. Because of the orbital symmetry of this Hamiltonian (the intra-orbital terms are the same for the two orbitals), and the restriction to only a local intra-orbital Hubbard interaction, one may also regard this model as a bilayer Hubbard model with layers $\alpha = 1, 2$.[1] The kinetic energy term $\mathcal{H}_{\mathcal{K}}$ can then be diagonalized and rewritten in terms of a bonding $k_z = 0$ and anti-bonding $k_z = \pi$ basis.

Throughout, we use $t = 1$ as the unit of energy and vary $U = 8t$, $t_{\perp}$, and the filling n to control the electronic structure of the system. We simulated Eq. (1) using the dynamical cluster approximation (DCA) method, where the bulk lattice system is mapped onto a periodic finite-size cluster embedded in a mean-field. The effective cluster problem was solved self-consistently by means of a continuous-time auxiliary field (CTAUX) quantum Monte Carlo method.[2–4]

The model's electronic structure is summarized in Fig. S1, which was obtained by extracting the single particle spectral function $A(\mathbf{k}, \omega)$ from the QMC data using the Maximum Entropy (MaxEnt) method.[5] The first plot in the Fig. S1 shows the electronic structure of the model with $U = 8t$, $t_{\perp} = 2.0t$, and a filling $n = 1.05$. For this choice of parameters we

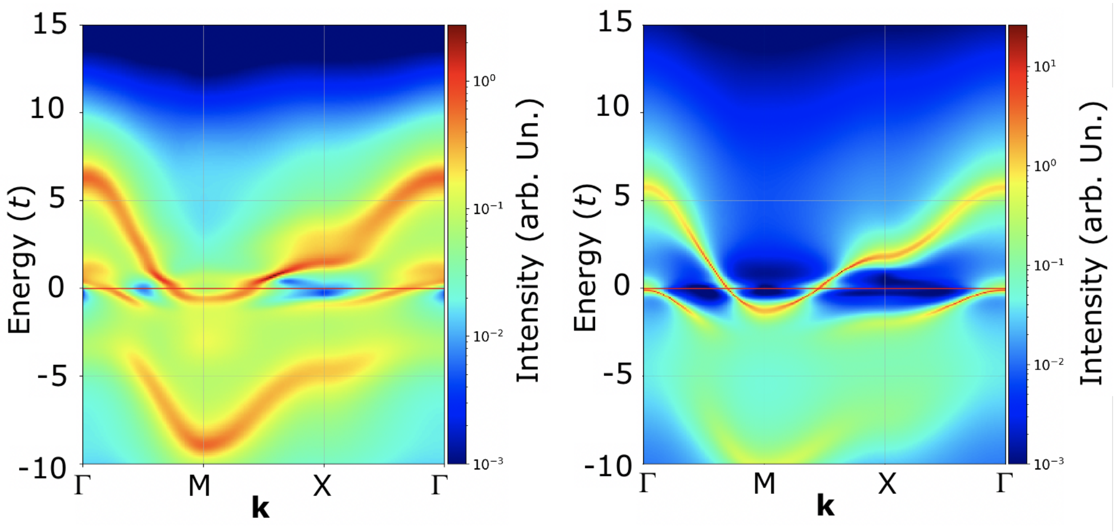

FIG. S1: Left: $A(\mathbf{k},\omega)$ obtained by setting $U/t = 8$, $t_\perp = 2$, $n = 1.05$ at $T = 0.2$ on a 4×4 lattice. In this case, both the electron-like band at the M point and the hole-like band at the Γ point cross the Fermi level. Right: the single particle spectral function $A(\mathbf{k},\omega)$, obtained by setting $U/t = 8$, $t_\perp = 2.5$, $n = 1.15$ at $T = 0.125$ on a 4×4 lattice. In this case, the Fermi surface has an electron-like pocket at the M point and an incipient hole-like band near the Γ point.

obtain a band structure that qualitatively resembles that of bulk FeSe where the hole band lies above the Fermi level at the Γ point, as shown in the right panel of Fig. S1. In contrast, for $t_\perp = 2.5t$, $U = 8t$, and $n = 1.15$, we obtain a band structure qualitatively similar to FeSe/STO. Specifically, we observe an electron pocket at the M point, $\mathbf{k}_\parallel = (\pi,\pi)$, resulting from the bonding $\mathbf{k}_z = (0,0)$ band and an incipient hole band with $k_z = \pi$ lying slightly below the Fermi level at the Γ point ($\mathbf{k}_\parallel = 0$).

To determine the magnetic excitation spectrum, we calculated the dynamical spin susceptibility

$$\chi^s_{\alpha,\beta}(\mathbf{r}_i - \mathbf{r}_j,\tau) = \langle \mathcal{T}_\tau S^z_{i,\alpha}(\tau) S^z_{j,\beta}(0)\rangle, \tag{2}$$

where $S^z_{i,\alpha}(\tau) = \frac{1}{2}\left[n_{i,\alpha,\uparrow}(\tau) - n_{i,\alpha,\downarrow}(\tau)\right]$ is the z-component of the spin on orbital α at site $\mathbf{r}_i$ at time τ.

Additionally, to investigate the charge excitations, we calculated the dynamical charge susceptibility

$$\chi^c_{\alpha,\beta}(\mathbf{r}_i - \mathbf{r}_j,\tau) = \langle \mathcal{T}_\tau n_{i,\alpha}(\tau) n_{j,\beta}(0)\rangle, \tag{3}$$

where $n_{i,\alpha}(\tau) = \frac{1}{2}[n_{i,\alpha,\uparrow}(\tau) + n_{i,\alpha,\downarrow}(\tau)]$ is the density on orbital α at site $\mathbf{r}_i$ at time τ. A Fourier transform to reciprocal space and Matsubara frequencies gives the momentum and frequency dependent spin and charge susceptibilities

$$\chi_{s/c}(\mathbf{Q}, i\omega_m) = \sum_{\mathbf{r},\alpha,\beta} \int_0^\beta d\tau\, \chi^{s/c}_{\alpha,\beta}(\mathbf{r},\tau) e^{i\mathbf{Q}_\parallel \mathbf{r}} e^{iQ_\perp(\alpha-\beta)} e^{i\omega_m \tau} \,. \tag{4}$$

Here, $\mathbf{Q} = (\mathbf{Q}_\parallel, Q_\perp)$ has both an intra-band component $Q_\perp = 0$ that scatters within the bonding $k_z = 0$ and anti-bonding $k_z = \pi$ bands, respectively, and an inter-band component $Q_\perp = \pi$ that scatters between the bands. As with the spectral functions, we obtained the dynamical susceptibilities by analytically continuing the spin and charge susceptibilities to the real axis using MaxEnt.

Figures S2 and S3 show the frequency and temperature dependence of the imaginary part of the spin and charge susceptibilities $\chi''_s(\mathbf{Q},\omega)$ and $\chi''_c(\mathbf{Q},\omega)$ for the case with two Fermi pockets and the case with an incipient band, respectively. In both figures, the left panel presents results for the intra-band component whereas the right panel shows results for the inter-band component of the momentum scattering vectors. In both cases, the amplitudes of the susceptibilities are largest for the inter-band scattering ($Q_\perp = \pi$) when compared to the intra-band case ($Q_\perp = 0$). In the first case, where the model has two Fermi pockets (Fig. S2), we observe a prominent peak that disperses to zero energy as the temperature decreases for the momentum scattering vector with components $\mathbf{Q}_\parallel = (\pi,\ \pi)$ and $Q_\perp = \pi$ in the spin channel. This behavior is due to the perfect nesting between the bonding and anti-bonding bands. Conversely, the spin excitations at this scattering vector are gapped when the hole pocket lies below the Fermi level, as in the incipient band case (Fig. S3). Whereas we observe gapped excitations in the charge channel shifted to the higher frequencies compared to the spin channel.

To provide evidence for the different types of dispersion between the two-band and incipient band models, we show another set of results for a different parameter regime demonstrating that our findings are not parameter dependent. In the first plot in Fig. S4, we report the electronic structure of the model with $U = 6t$, $t_\perp = 2.2t$, and a filling $n = 1.05$. The obtained band structure resembles the plot in Fig. S1 representing well the bulk FeSe in which there is an electron pocket at the M point and hole pocket at the Γ point. In the right plot of Fig. S4, the electronic structure of the model with $U = 6t$, $t_\perp = 2.8t$, and a filling

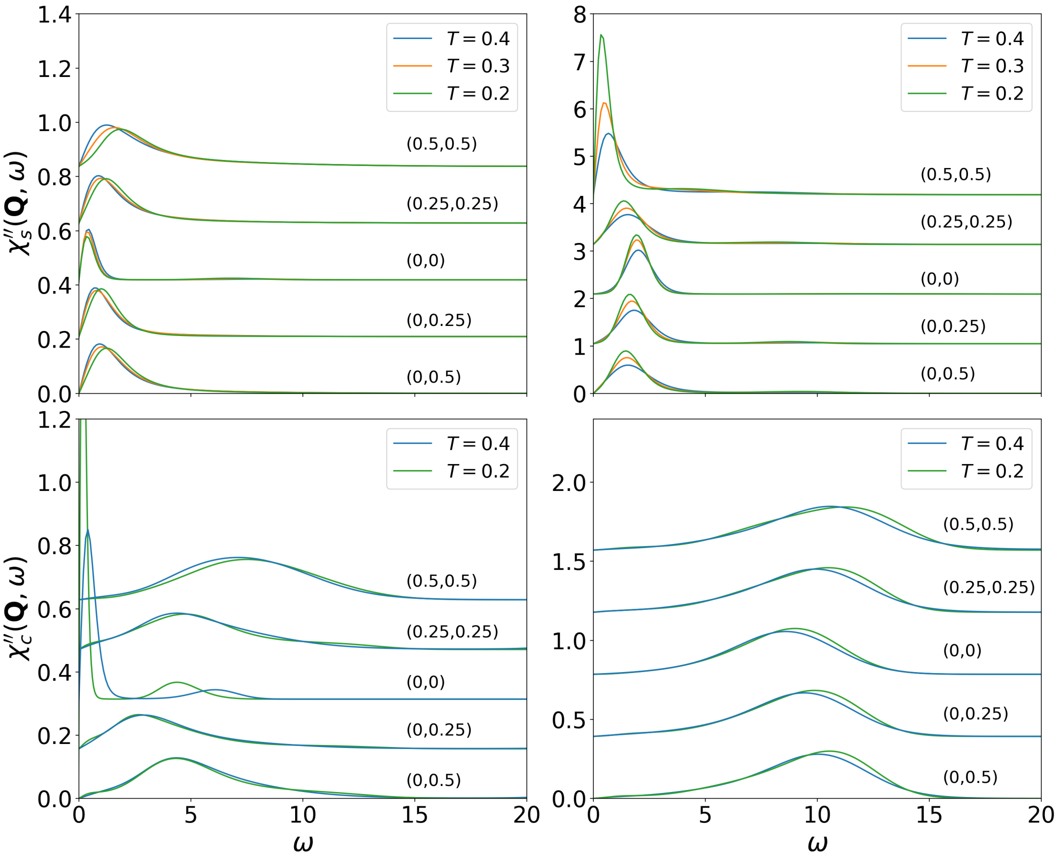

FIG. S2: Two band model. Frequency dependence of charge and spin susceptibility $\chi''_{c,s}(\mathbf{Q},\omega)$ along high symmetry directions for different temperatures at $U/t = 8$. Results are shown here for the case with two Fermi pockets. On the top row we report the spin susceptibility and on bottom row the charge susceptibility. We report the calculations for $Q_\perp = 0$ or intra-band (left panel) and $Q_\perp = \pi$ or inter-band (right panel).

$n = 1.15$ is shown. In Fig. S4, we observe an electron pocket at the M point and an incipient hole band lying slightly below the Fermi level at the Γ point representing the FeSe/STO. From this parameters set, we have also calculated the spin and charge susceptibilities which are presented in the figures S5 and S6. We observe the same qualitative behaviours (acoustic spin susceptibility in the two band model vs optical spin susceptibility in the incipient band case) of the spin and charge susceptibilities both for the bulk FeSe and the FeSe/STO as in the first data set presented above.

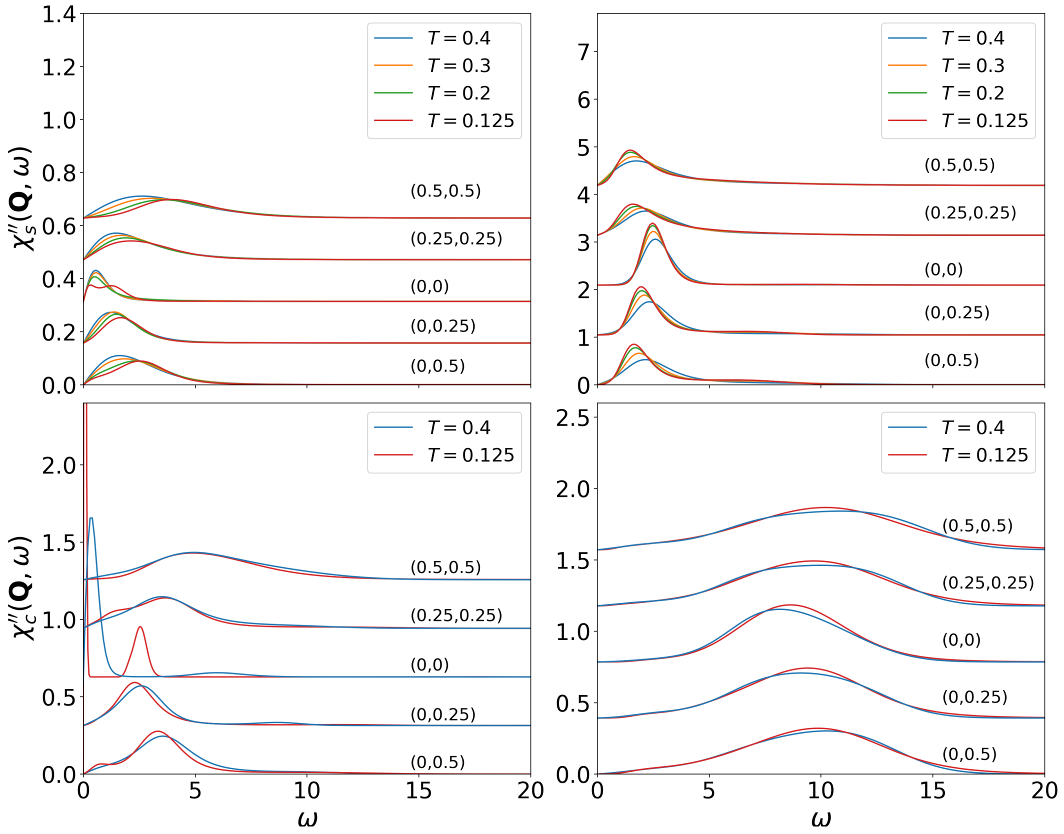

FIG. S3: Incipient band model. Frequency dependence of charge and spin susceptibility $\chi''(\mathbf{Q},\omega)$ along high symmetry directions for different temperatures at $U/t = 8$. Results are shown here for the incipient band case. On the top row we report the spin susceptibility and on bottom row the charge susceptibility. We report the calculations for $Q_\perp = 0$ or intra-band (left panel) and $Q_\perp = \pi$ or inter-band (right panel).

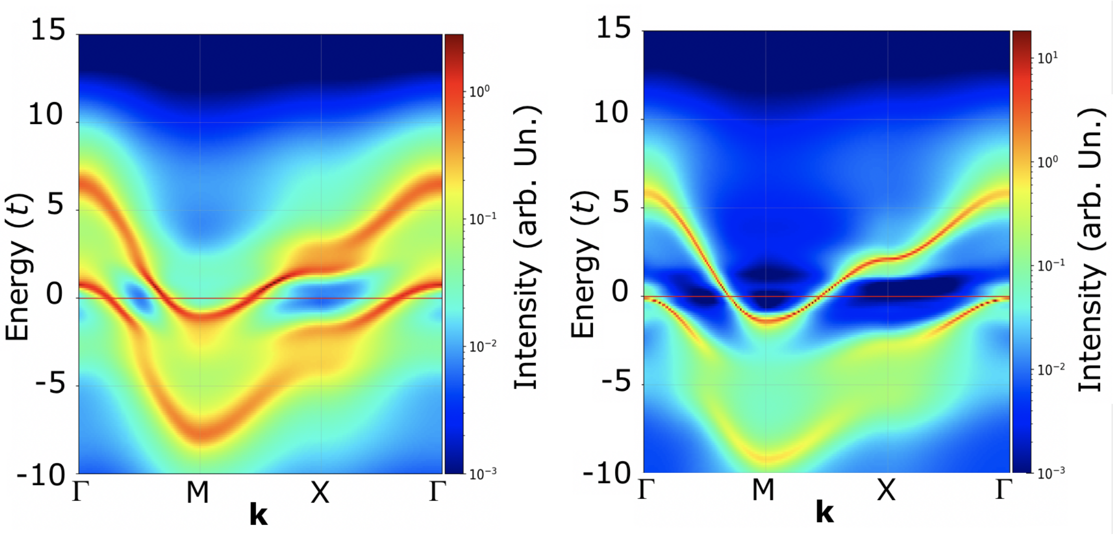

FIG. S4: Left: $A(\mathbf{k}, \omega)$ obtained by setting $U/t = 6$, $t_{\perp} = 2.2$, $n = 1.05$ at $T = 0.2$ on a 4×4 lattice. In this case, both the electron-like band at the M point and the hole-like band at the Γ point cross the Fermi level. Right: single particle spectral function $A(\mathbf{k}, \omega)$, obtained by setting $U/t = 6$, $t_{\perp} = 2.8$, $n = 1.15$ at $T = 0.2$ on a 4×4 lattice. In this case, the Fermi surface has an electron-like pocket at the M point and an incipient hole-like band near the Γ point.

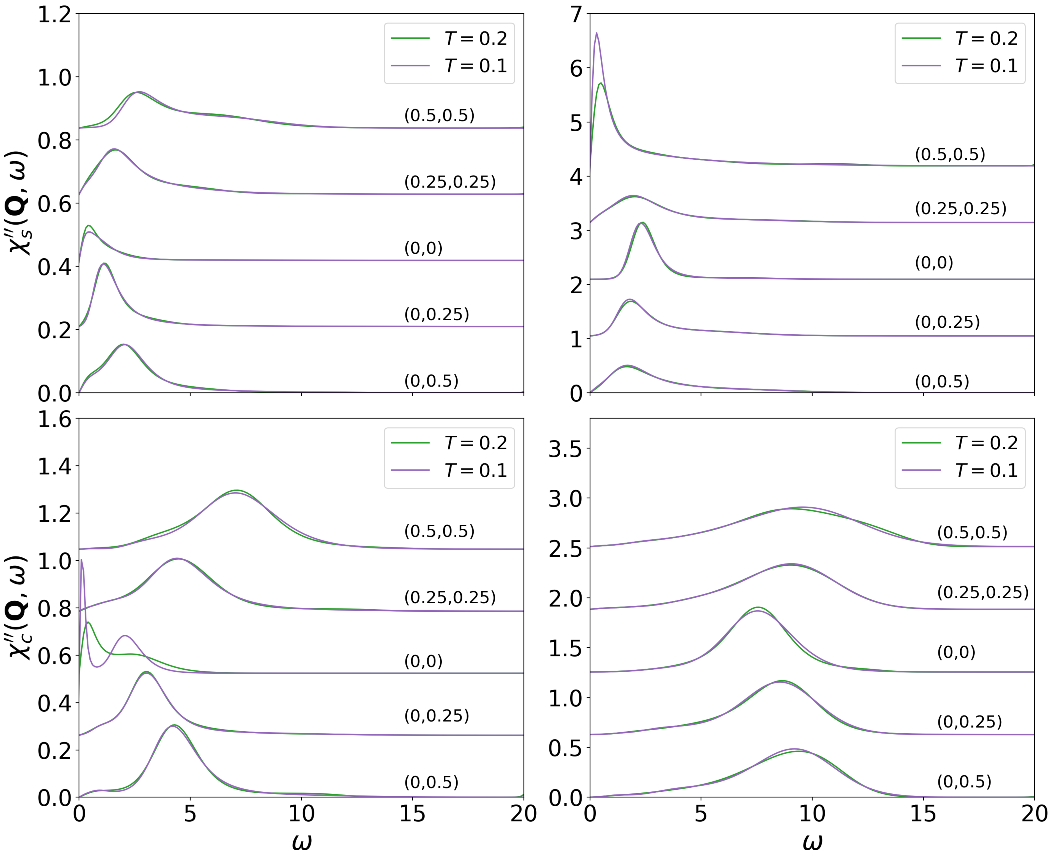

FIG. S5: Frequency dependence of the susceptibility $\chi''(\mathbf{Q},\omega)$ of the two band model along high symmetry directions for different temperatures at $U/t = 6$. On the top row we report the spin susceptibility and on bottom row the charge susceptibility. We report the calculations for $Q_\perp = 0$ or intra-band (left panel) and $Q_\perp = \pi$ or inter-band (right panel).

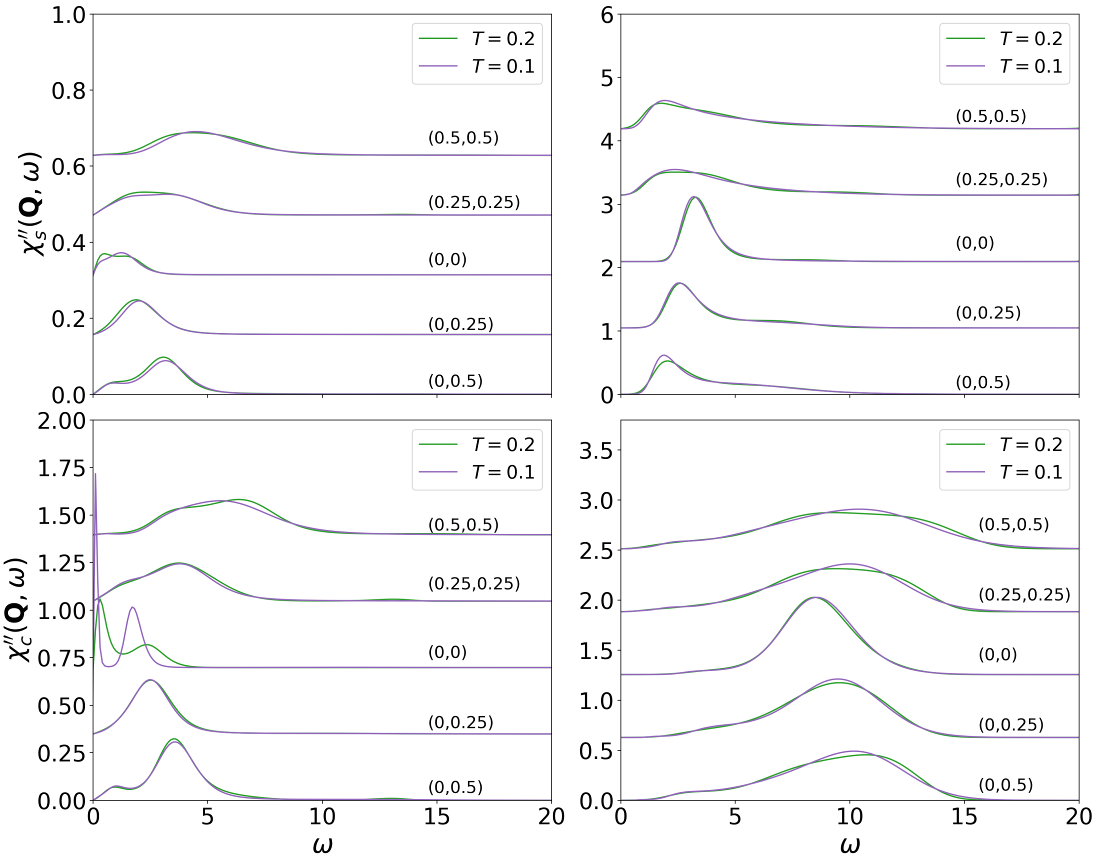

FIG. S6: Frequency dependence of the susceptibility $\chi''(\mathbf{Q},\omega)$ of the incipient band model along high symmetry directions for different temperatures at $U/t = 6$. On the top row we report the spin susceptibility and on bottom row the charge susceptibility. We report the calculations for $k_\perp = 0$ or intra-band (left panel) and $k_\perp = \pi$ or inter-band (right panel).

II. A SIMPLIFIED LADDER MODEL

To verify the robustness of the interpretation of our 2D bilayer model results, we additionally performed exact diagonalization calculations of a small 6×2 Hubbard ladder with periodic boundary conditions. This system is a 1D analog of the model considered in the main text, and is small enough that we can explicitly compute the RIXS intensity with reasonable momentum resolution.

The Hamiltonian of the 1D Hubbard ladder is given by

$$H = -t \sum_{l,\alpha,\sigma} \left(c^{\dagger}_{l,\alpha,\sigma} c_{l+1,\alpha,\sigma} + \text{H.c.} \right) - t_{\perp} \sum_{l,\sigma} \left(c^{\dagger}_{l,0,\sigma} c_{l,1,\sigma} + \text{H.c.} \right) + U \sum_{l,\alpha} \hat{n}_{l,\alpha,\uparrow} \hat{n}_{l,\alpha,\downarrow}, \quad (5)$$

where $c^{\dagger}_{l,\alpha,\sigma}$ ($c_{l,\alpha,\sigma}$) creates (annihilates) a spin σ ($=\uparrow,\downarrow$) hole on leg α ($= 0, 1$) of rung l, t is the nearest-neighbor hopping integral along the leg, $t_{\perp}$ is the nearest-neighbor along the rung, and U is the on-site Hubbard repulsion.

To understand the model's dynamical properties, we computed its single-particle spectral function $A(k, \omega)$, dynamical spin structure factor $S(\mathbf{q}, \omega)$, x-ray absorption spectra $I_{\text{XAS}}(\omega)$, and RIXS intensity $I_{\text{RIXS}}(\mathbf{q}, \omega)$ in the $\Delta S = 1$ channel, which dominates the RIXS spectra at the transition metal L-edge. As in the bilayer model, we can partition $\mathbf{q} = (q_{\|}, q_{\perp})$, where $q_{\|}$ is the momentum transfer along the ladder leg and $q_{\perp} = 0$ and π selects the intra- and interband scattering processes.[12] Throughout this section, we work in electron language and diagonalize Eq. (5) in the ($n_{\uparrow} = 7, n_{\downarrow} = 6$) sector to determine the ground state $|g\rangle$, which is equivalent to an electron doping of ≈ 1.08 electrons per orbital. The model parameters are $t = 1$, $U = 4t$, and $t_{\perp} = 3t/2$, $2t$, and $3t$.

A. The single-particle spectral function

We begin by examining the single-particle spectral function. This quantity can be divided into contributions from electron addition (+) and removal (−) processes $A(k, \omega) = A_{+}(k, \omega) + A_{-}(k, \omega)$, with

$$A_{\pm}(k, \omega) = \sum_{f,\alpha,\sigma} |\langle f| c^{\pm}_{k,\alpha,\sigma} |g\rangle|^2 \delta(E_f - E_g - \omega) \quad (6)$$

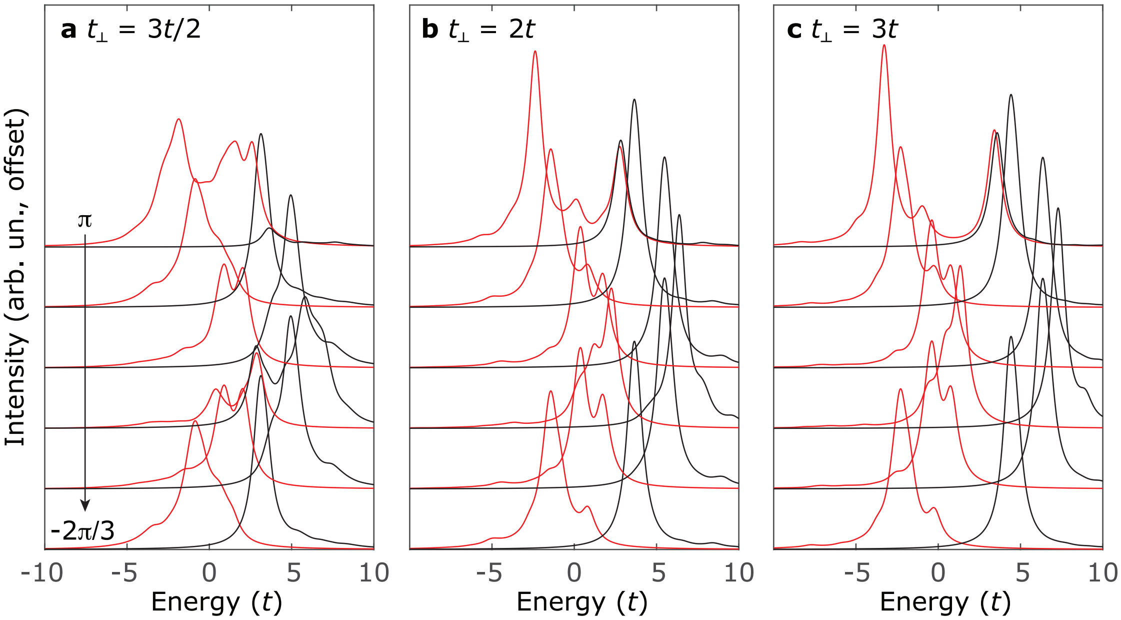

FIG. S7: Single-particle spectral function of the ladder model. The single-particle spectral function $A(k,\omega)$ for the doped ladders, divided into their addition (black lines) and removal (red lines) spectra.

Here, $|g\rangle$ and $|f\rangle$ are the ground and final states with energy E_g and E_f, respectively, and

$$c^{+}_{k,\alpha,\sigma} \equiv c^{\dagger}_{k,\alpha,\sigma} = \frac{1}{\sqrt{N}} \sum_{l} e^{-\mathrm{i}kR_l} c^{\dagger}_{l,\alpha,\sigma},$$

$$c^{-}_{k,\alpha,\sigma} \equiv c_{k,\alpha,\sigma} = \frac{1}{\sqrt{N}} \sum_{l} e^{-\mathrm{i}kR_l} c_{l,\alpha,\sigma},$$

where R_l is the position of rung l along the ladder direction.

Results for the addition (black lines) and removal (red lines) spectra are plotted in Fig. S7(a)-(c) for $t_\perp = 3t/2$, $2t$, and $3t$, respectively. In the absence of any interactions, the non-interacting bands are $\epsilon_\pm = -2t\cos(ka) \mp 2t_\perp$. We would then expect to have one band completely occupied for a slightly electron-doped system while the second band only has its states at lowest energies filled. Moreover, this band should be located approximately $4t_\perp$ above the first. While a non-zero $U = 4t$ will further dress the electronic structure, we have found that this non-interaction picture provides a useful framework for understanding our results.

Figure S7(c) shows results for $A(k,\omega)$ with $t_\perp = 3t$. Here, we see two dispersing bands. The first band consists of fully occupied states located at energies $\omega < 3t$. The second is located at higher energies and has nearly degenerate electron removal and addition states

located at its band bottom centered at $q = \pi$. Decreasing $t_\perp$ to $2t$ produces a qualitatively consistent spectral function but pushes the two bands closer to one another, as shown in Fig. S7(b). As $t_\perp$ is decreased further to $3t/2$, the first removal state at $q = 0$ becomes degenerate with an electron addition state. This observation reflects the fact that the top of the lower-energy band now lies above the Fermi level. These results show that $t_\perp = 3t/2$ produces two bands crossing the Fermi level, with hole-like and electron-like bands centered at $q = 0$ and π, respectively. Conversely, $t_\perp \geq 2t$ produces an incipient band scenario, where one band is filled, and the other has a small electron-like Fermi surface centered at the zone boundary. These cases are analogous to the 2D results shown in the main text.

B. The RIXS response and its comparison with the dynamical spin structure factor

Next, we turn our attention to the RIXS response, which is computed within the full Kramers-Heisenberg formalism. Specifically, the RIXS intensity is given by

$$I_{\text{RIXS}}(q_\parallel, \omega) = \sum_f \left| \sum_n \frac{\langle f| D^\dagger_{\mathbf{k}_{\text{out}}} |n\rangle \langle n| D_{\mathbf{k}_{\text{in}}} |g\rangle}{E_g + \omega_{\text{in}} - E_n + \text{i}\Gamma} \right|^2 \delta(E_f - E_g + \omega), \tag{7}$$

where $|g\rangle$, $|n\rangle$, and $|f\rangle$ are the initial, intermediate, and final states of the scattering process, respectively, E_g, E_n, and E_f are their corresponding energies, $\mathbf{k}_{\text{in}}$ and ω_{in} ($\mathbf{k}_{\text{out}}$ and ω_{out}) are the momentum and energy of the incoming (outgoing) photons, $q_\parallel = (\mathbf{k}_{\text{out}} - \mathbf{k}_{\text{in}})_\parallel$ and $\omega = \omega_{\text{out}} - \omega_{\text{in}}$ are the momentum and energy transferred to the ladder during the RIXS scattering process, $\Gamma = t/2$ is related to the core-hole lifetime, $D_l = \sum_{\alpha,\sigma} P_\alpha e^{\text{i}q_\parallel R_l} c^\dagger_{l,\alpha,\sigma} p_{l,\sigma}$ is a local dipole operator responsible for the $2p \to 3d$ transition and $p_{l,\sigma}$ annihilates an electron in the $2p$ core level. The phase factor P_α in the definition of the dipole operator is used to isolate the intra- and inter-band contributions to the scattering. For intra-band scattering $P_0 = P_1 = 1$ while for inter-band scattering $P_0 = -P_1 = 1$. When diagonalizing the Hamiltonian to obtain the intermediate states $|n\rangle$, we also add a local potential

$$H_{\text{ch}} = U_q \sum_{l,\alpha,\sigma} \hat{n}_{l,\alpha,\sigma}[1 - \hat{n}^p_{l,\sigma}] \tag{8}$$

to Eq. (5), which accounts for the attractive potential produced by the core hole. Here, $\hat{n}^p_{l,\sigma} = p^\dagger_{l,\sigma} p_{l,\sigma}$ is the number operator for the core hole on rung l and we set $U_q = -2t$.

All of our RIXS calculations are carried out with an incident photon energy tuned to the maximum in the XAS spectra. The XAS spectra are given by

$$I_{\rm XAS}(\omega) = \sum_n |\langle n| D_{\mathbf{k}_{\rm in}=0} |g\rangle|^2 \delta(E_n - E_g - \omega). \tag{9}$$

We also computed the dynamical spin structure factor

$$S(q,\omega) = \sum_f |\langle f| \hat{S}^z_{q,\pm} |g\rangle|^2 \delta(E_f - E_g - \omega) \tag{10}$$

to help facilitate comparisons with our results shown in the main text, where the RIXS cross section cannot be computed on large clusters. Here, $\hat{S}^z_{q,\pm} = \frac{1}{\sqrt{2N}}\sum_l e^{-{\rm i}qR_l}(\hat{S}^z_{l,1} \pm \hat{S}^z_{l,0})$, where the + or (−) sign again corresponds to the intra- and inter-band components, and $\hat{S}^z_{l,\alpha} = \hat{n}_{l,\alpha,\uparrow} - \hat{n}_{l,\alpha,\downarrow}$ is the spin-z operator of the orbital on rung l and leg α.

At transition metal L-edges, the magnetic excitations are typically dominated by the $\Delta S = 1$ scattering channel. The intra- and inter-band components of the RIXS spectra in the $\Delta S = 1$ channel are shown in the top rows of Figs. S8 and S9, respectively. [The incident photon energy $\omega_{\rm in}$ in both cases was chosen to coincide with the maximum in the corresponding XAS spectra, as shown in the insets of Fig. S8, panels (a)-(c).] The corresponding intra- and inter-band components of the dynamical spin structure factors are shown in the bottom rows [panels (d)-(e)] of each figure.

In the intra-band scattering channel, the RIXS response has a dispersing feature emanating from $q = 0$ that originates from intra-band particle-hole excitations in the bands crossing the Fermi level. These excitations are most intense in the two-band case [Fig. S8(a)] and are suppressed in the incipient band cases [Figs. S8(b),(c)]. This change reflects the removal of the hole-like band from the Fermi surface in the latter case, which eliminates a channel for intra-band particle-hole excitations. This behavior is also reflected in the corresponding intra-band components of the dynamical structure factor, indicating that $S(q,\omega)$ provides a reasonable proxy for the low-energy magnetic RIXS response in our multi orbital toy model.

We obtain qualitatively different behavior in the inter-band response, shown in Fig. S9. In this case, the inter-band magnetic excitations have a continuum-like structure, similar to prior calculations for doped Hubbard ladders.[13] For the two-band case, shown in Fig. S9(a), the excitations are gapped at $q = 0$ and disperse towards lower energies at the zone boundaries. In the incipient band case, shown in Figs. S9(b) and S9(c), the same excitations are pushed to higher energies and are gapped by an amount proportional to $t_\perp$. As with the

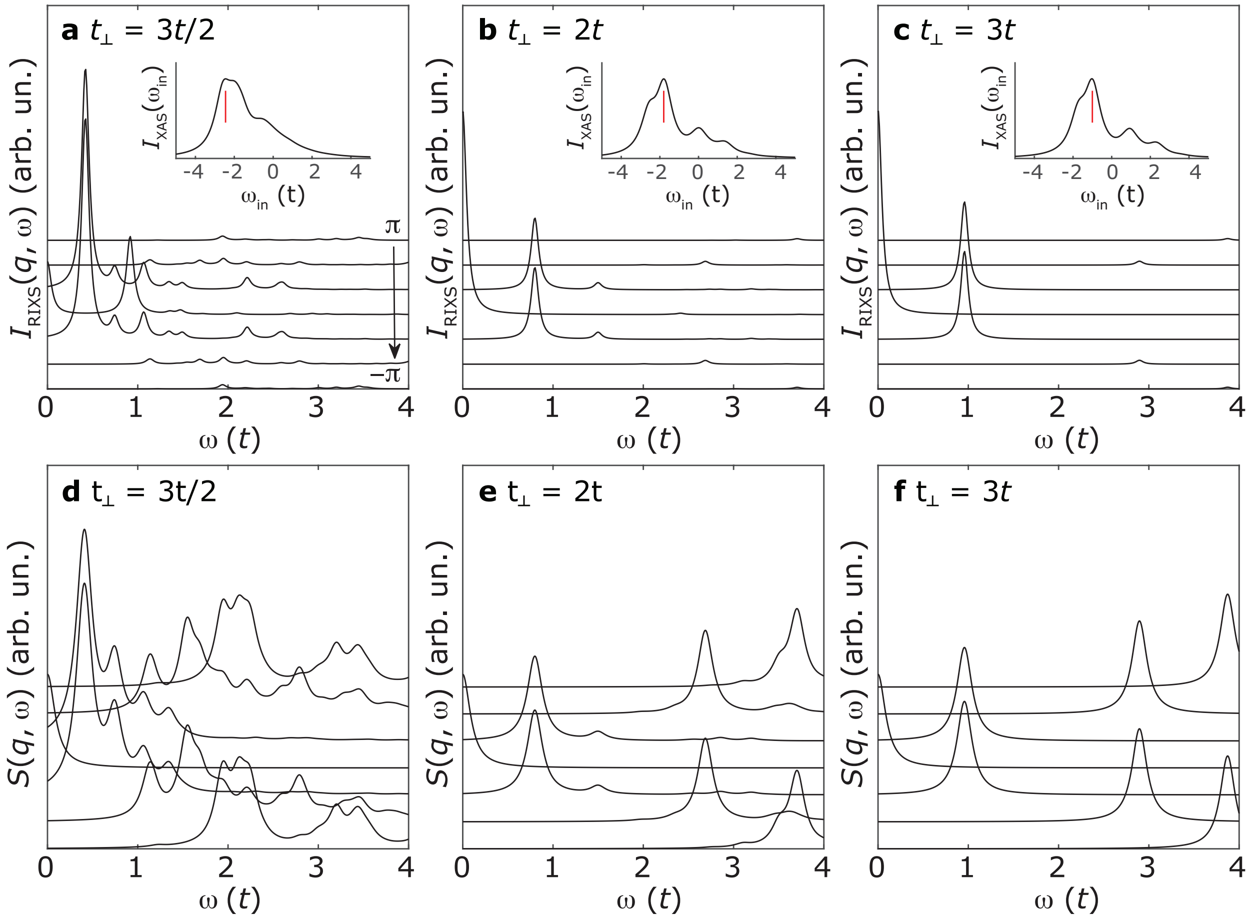

FIG. S8: Intra-band RIXS response. The top row plots the intra-band components ($q_y = 0$) of the RIXS response in the $\Delta S = 1$ channel, computed for (a) $t_\perp = 3t/2$, (b) $t_\perp = 2t$, and (c) $t_\perp = 3t$. The incident photon energy in each case was tuned to the resonance peak in the corresponding XAS spectra, as shown in the insets of each figure. The second row shows the intra-band scattering components of the dynamical spin structure factor for (d) $t_\perp = 3t/2$, (e) $t_\perp = 2t$, and (f) $t_\perp = 3t$. The RIXS spectra are plotted on the same relative intensity scale so the intensity of panels (a)-(c) can be meaningfully compared to each other. The same holds true for panels (d)-(e).

intra-band case, these features are qualitatively captured by the inter-band components of the dynamical spin structure factor, shown in Figs S9(d)-(e). All of the observations derived from this 1D toy model are consistent with those observed in our 2D bilayer model. Moreover, the qualitative consistency between the RIXS intensity and $S(q, \omega)$ provides theoretical support for the notion that the dynamical spin susceptibility can provide a qualitative description of the excitations probed by RIXS, as discussed in the main text.

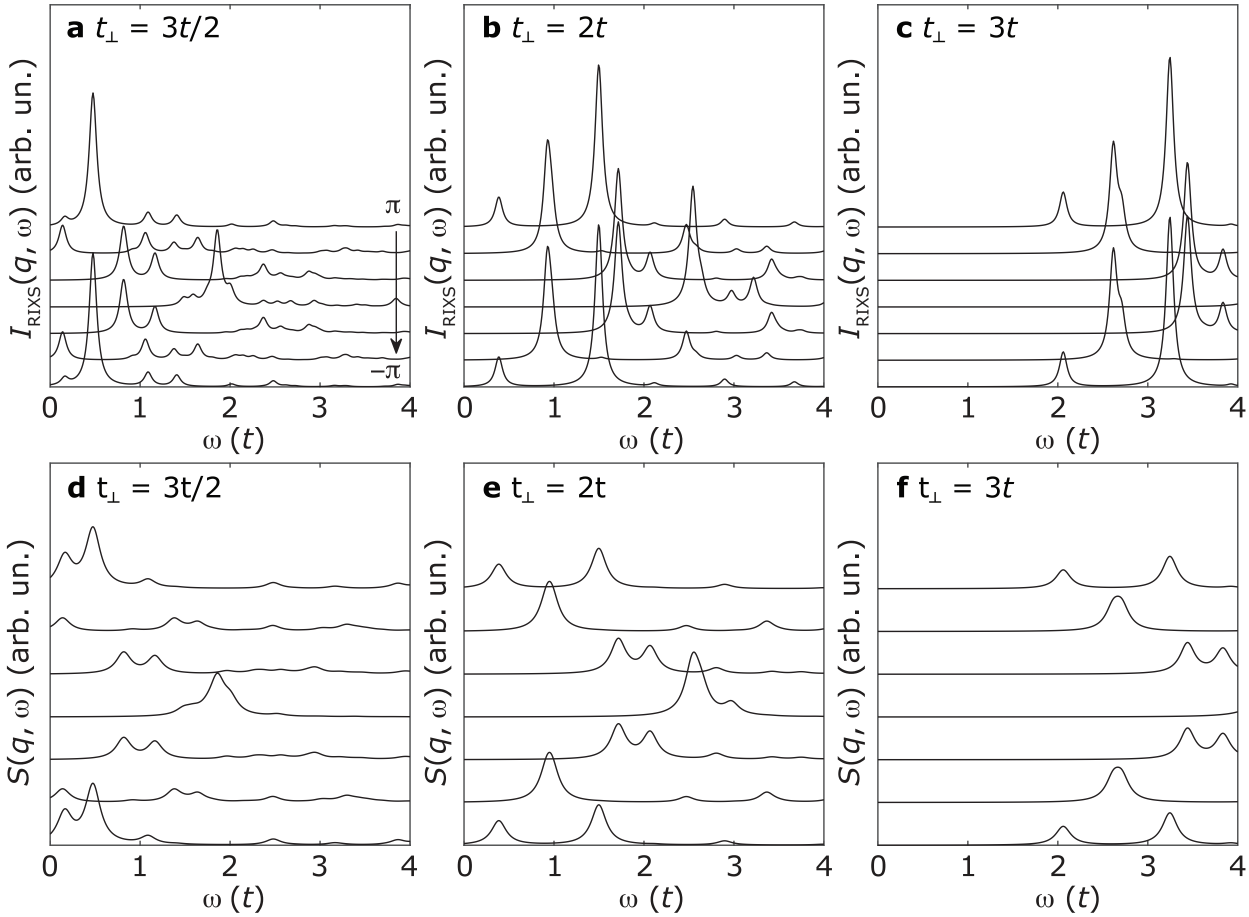

FIG. S9: Inter-band RIXS response. The top row plots the inter-band components ($q_y = \pi$) of the RIXS response in the $\Delta S = 1$ channel, computed for (a) $t_\perp = 3t/2$, (b) $t_\perp = 2t$, and (c) $t_\perp = 3t$. The incident photon energy in each case was tuned to the resonance peak in the corresponding XAS spectra, as shown in the insets of each figure. The second row shows the inter-band scattering components of the dynamical spin structure factor for (d) $t_\perp = 3t/2$, (e) $t_\perp = 2t$, and (f) $t_\perp = 3t$. The RIXS spectra are plotted on the same relative intensity scale so the intensity of panels (a)-(c) can be meaningfully compared to each other. The same holds true for panels (d)-(e).

III. RIXS DATA

A. Data along (H,H)

Figure S10 reports the RIXS data acquired from bulk FeSe along the (H,H) direction. Similarly to the (H,0) direction, we detect the presence of dispersive spin excitations moving

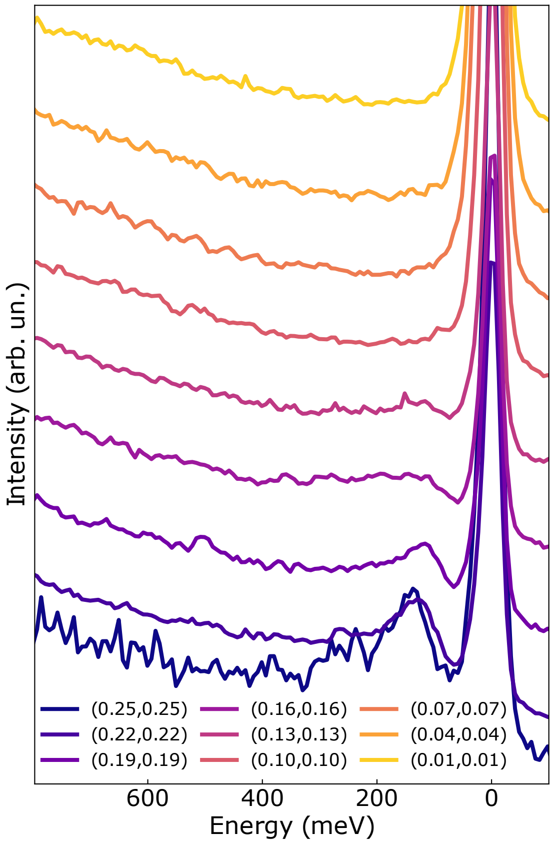

FIG. S10: RIXS data on FeSe bulk collected along the (H,H) direction.

to lower energies as we approach the zone center.

Figure S11 shows the RIXS spectra of FeSe/STO along the (H,H) direction. As with the data taken along the (H,0) direction, we observe a broad peak centered at 300-400 meV that hardly disperses in momentum space.

B. Energy detuning for FeSe/STO

As mentioned in the main text, spectroscopic measurements on a monolayer of materials are difficult to interpret. To ensure our sensitivity to the FeSe grown onto the STO substrate, we collected RIXS measurements at different incident energies and present the results in Fig. S12. As we tune the incident photon energy away from the maximum of the Fe absorption edge we observe a clear decrease of intensity. This observation corroborates the hypothesis that the RIXS signal originates from FeSe and not from the substrate or capping layer.

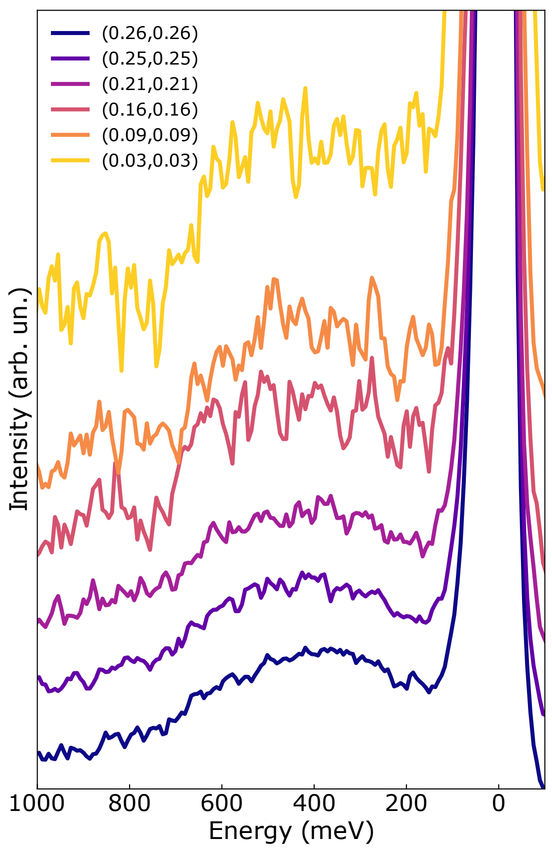

FIG. S11: RIXS data on FeSe/STO collected along the (H,H) direction.

The peak that we ascribed to the spin excitation (see main text for a detailed discussion) of FeSe/STO displays a Raman-like behaviour as the energy is changed. The peak energy does not change with the incident energy, an observation which signals that we are coherently probing an excitation of the system and not fluorescent emission (which would be at fixed emission energy, and not at fixed energy transfer). At the same time, increasing the incident energy strongly suppresses the peak observed at low energy loss.

C. Fitting of RIXS spectra in FeSe bulk and monolayer

1. *Fitting of bulk FeSe*

We fit the RIXS spectra of the FeSe bulk using a variation of the method employed in previous papers.[6–11] The model consists of two guassian curves accounting for the elastic line, and a phonon at 20-30 meV, an asymmetric lorentzian for the spin excitations, and a

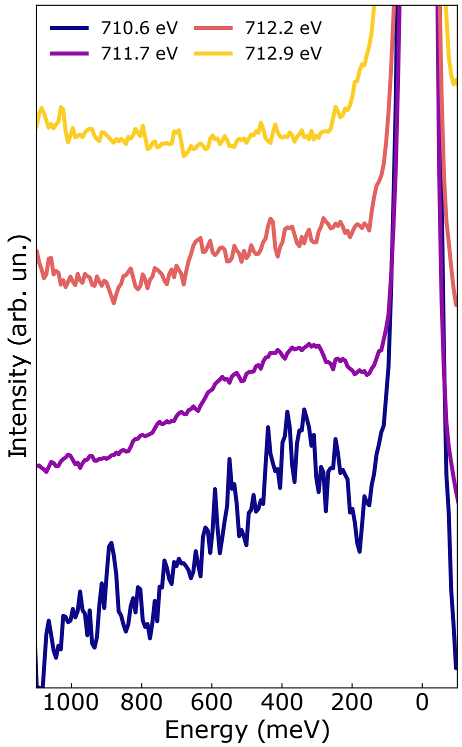

FIG. S12: RIXS data on FeSe/STO collected at different incident energies.

polynomial background. The results are reported for a single **Q** point in Fig. S13**a**.

2. *Fitting of the FeSe/STO*

For the case of FeSe/STO we performed a fitting using two gaussian curves at low energy (elastic and phonon), and an asymmetric lorentzian for the spin excitations feature. The background is less intense and could be neglected in this sample. In Fig. S13**b**, we show the fitting for the case of the FeSe/STO. The spectral weight in the region 50-150 meV is not perfectly reproduced. The presence of a strong elastic line due to diffuse scattering from the interfaces of the sample could lead to a broadening of the elastic line which could not reliably be fitted. The non-fitted extra spectral weight could also be due to saturation of some of the pixels of the CCD due to the strong elastic signal (with intensity leaking on either sides of the elastic peak). In any case, a more involved fitting achieved by adding an additional gaussian would not produce any additional information or affect the peak position of the spin excitations peak discussed in the main text.

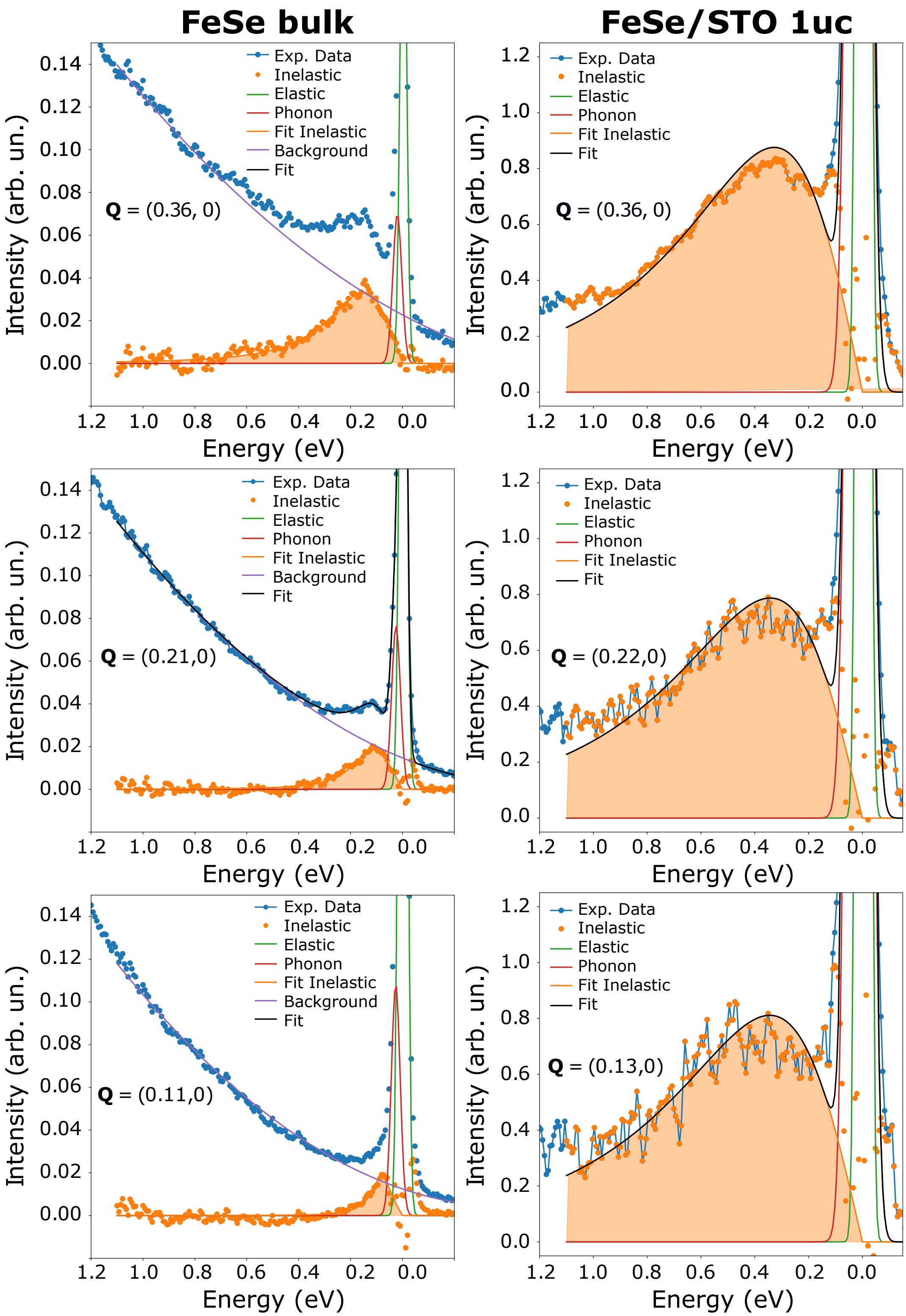

FIG. S13: Representative fitting for the bulk and monolayer FeSe at high, medium and low momentum transfer.

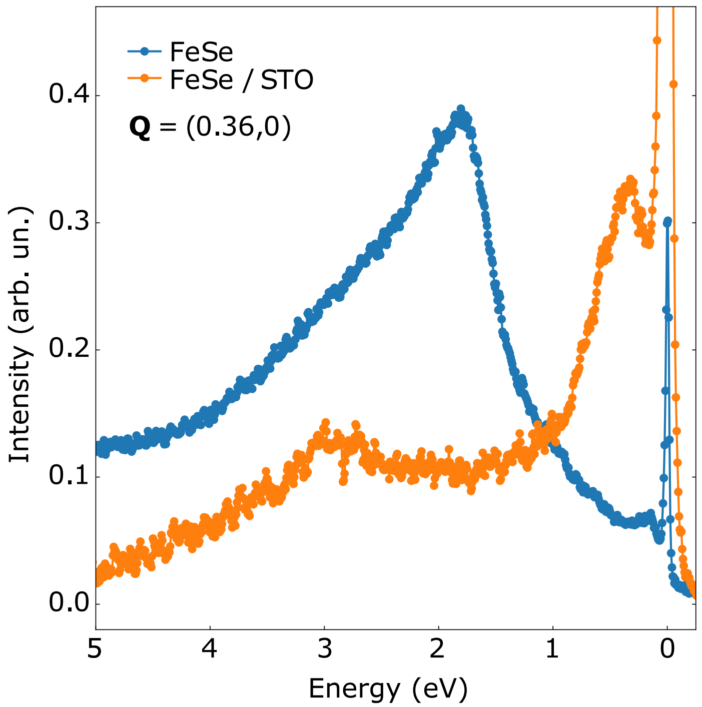

FIG. S14: Comparison at high energy loss of FeSe and FeSe/STO at $\mathbf{Q} = (0.36, 0)$.

3. Comparison of high energy loss

In Fig. S14 we display a comparison between FeSe and FeSe / STO at high energy loss up to 5 eV. The spectral intensity has been adjusted for better visualization. The fluorescence line of FeSe resembles that of Fe pnictides, but the lineshape of FeSe/STO is highly distorted by the limited thickness. For a more exhaustive comparison, measurements with higher statistics are needed.

IV. COMPARISON OF RIXS DATA WITH SUSCEPTIBILITY

In Fig. S15 we report a comparison of charge susceptibility and the RIXS experimental data for FeSe/STO and the incipient band model **a** and FeSe bulk and the two band model **b** for different momenta. We include both intra- (orange) and interband (blue) charge susceptibility. To convert the energy from units of t to energy we used the same values reported above for the spin susceptibility (90 and 160 meV for the bulk and incipient band cases). In both models the interband charge susceptibility peaks at much higher energy than the interband and shows a flat gapped disspersion. The intraband susceptibility (blue

traces) presents a dispersion qualitatively similar to the spin component but much steeper. In both incipient and two band cases the calculations of the charge susceptibility do not agree with the experimental data.

In Fig. S16 we report a comparison of the sum of the spin and charge susceptibility with the experimental data at different momenta. We indicate as orange trace the interband component and as blue trace the intraband one. In this figure we observe the dominance of the interband susceptibility over the intraband one. In the case of the incipient band comparison this leads to a pretty good agreement between the interband susceptibility (dominated by the spin component) and the experimental data. For the case of the two band the model the agreement of the interband susceptibility is poor compared to the experimental data due to the lack of dispersion. However a good agreement between the intraband susceptibiliy (dominated by the spin component) is observed.

In Fig. S17, we display as a color plot the charge susceptibility as a function of Q and energy and the experimental data. In panels **a,c (b,d)** we report the intraband and interband charge susceptibility for the two band model (incipient band model). As white dots and diamonds we report the experimental data for the monolayer and bulk cases, respectively. We used the same t parameters of Fig. 3 of the main text to rescale the energy. Contrary to the plot of the spin susceptibility we observe a poor agreement between the charge dispersion and our experimental data, proofing that RIXS our experimental observations are connected to the spin components rather than the charge one.

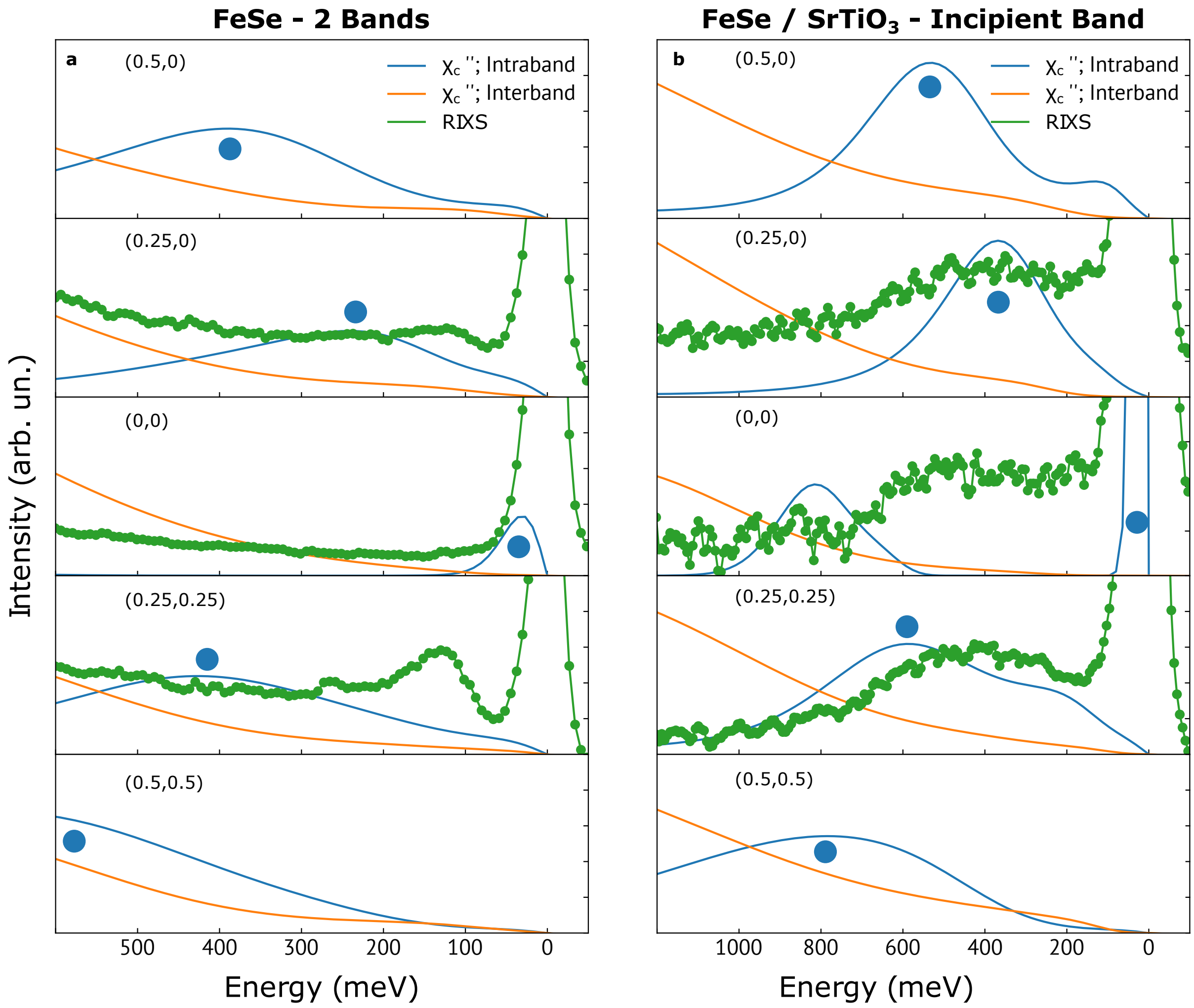

FIG. S15: Comparison of charge susceptibility and RIXS data. Left (right) panel: FeSe bulk (FeSe/STO) RIXS data and $\chi_c''(\mathbf{q},\omega)$ from a two bands (incipient) band model for intra- (blue line) and interband (orange line) charge susceptibility. Blue circles indicate the energy position of the maximum of the spin susceptibility in the intraband cases.

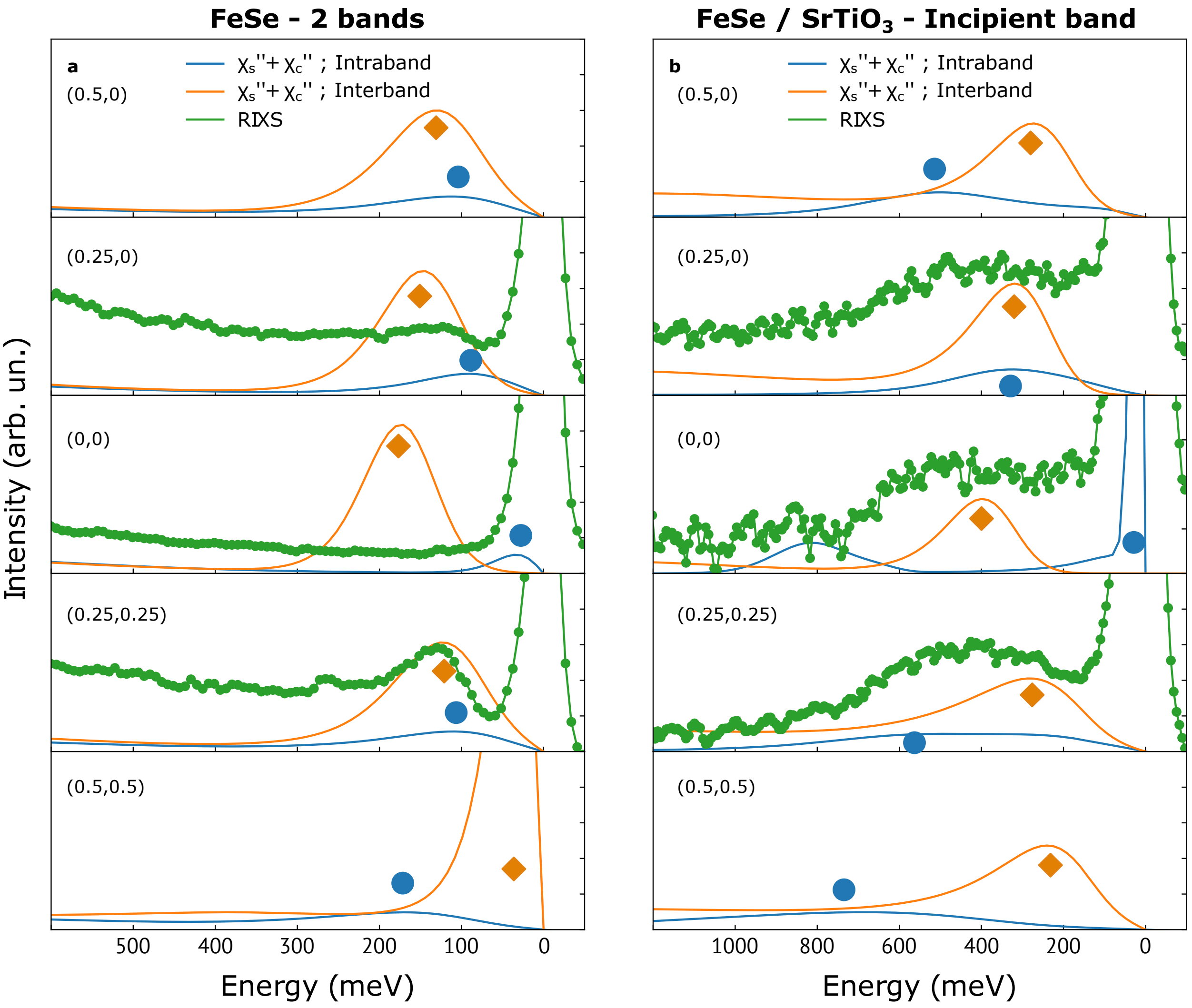

FIG. S16: Comparison of total susceptibility (charge and spin) susceptibility and RIXS data. Left (right) panel: FeSe bulk (FeSe/STO) RIXS data and $\chi''_{tot}(\mathbf{q},\omega)$ from a two bands (incipient) band model for intra- (blue line) and interband (orange line) charge susceptibility. Orange circles (blue) indicate the energy position of the maximum of the spin susceptibility in the interband (intraband) cases.

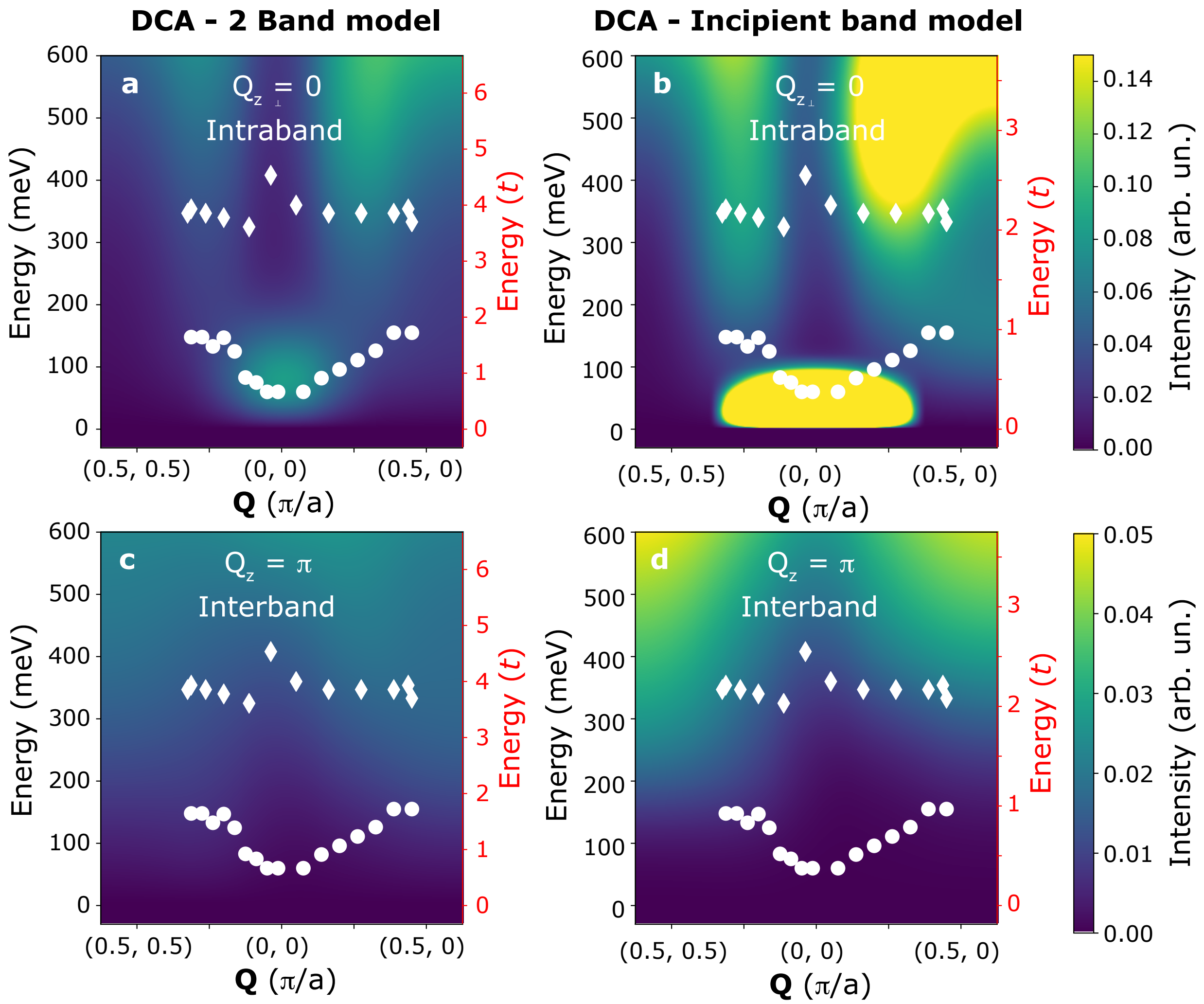

FIG. S17: Dynamical Charge susceptibility from DCA calculations. **a-d**: DCA calculations of the imaginary part of the charge susceptibility $\chi_c''(\mathbf{q},\omega)$ for the two-band Hubbard model (**a**: intraband $Q_z = 0$; **c**: interband $Q_z = \pi$) and the incipient band Hubbard model (**b**: intraband $Q_z = 0$; **d**: interband $Q_z = \pi$). Red circles (white diamonds) indicate the energy position of the peak detected by RIXS in bulk (monolayer) FeSe. The uncertainties associated with peak fitting are smaller than the markers.

V. SUPERCONDUCTING GAP OF FESE/STO

The T_c of the FeSe/STO has been characterized by ARPES before applying the capping layer of amorphous Se. In Fig. S18 we show ARPES data of the superconducting gap. The gap has been estimated to be in 13.7 meV, corresponding to a $T_c \approx 60 - 65$ K.

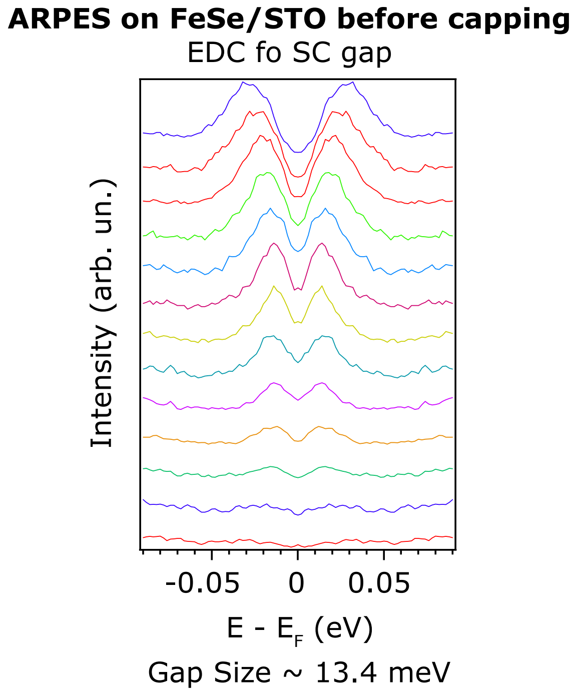

FIG. S18: ARPES measurements of superconducting gap in FeSe/STO before capping.

* email: pelliciari@bnl.gov

† email: rcomin@mit.edu

[1] Maier, T. A. & Scalapino, D. J. Pair structure and the pairing interaction in a bilayer Hubbard model for unconventional superconductivity. *Physical Review B* **84**, 180513 (2011). URL `https://link.aps.org/doi/10.1103/PhysRevB.84.180513`.

[2] Hähner, U. R. *et al.* DCA++: A software framework to solve correlated electron problems with modern quantum cluster methods. *Computer Physics Communications* **246**, 106709 (2020). URL `http://www.sciencedirect.com/science/article/pii/S0010465519300086`.

[3] Gull, E., Werner, P., Parcollet, O. & Troyer, M. Continuous-time auxiliary-field Monte Carlo for quantum impurity models. *EPL (Europhysics Letters)* **82**, 57003 (2008). URL `http://stacks.iop.org/0295-5075/82/i=5/a=57003?key=crossref.a04bd39c153e80d2afe29b4a20da2527`.

[4] Gull, E. *et al.* Submatrix updates for the continuous-time auxiliary-field algorithm. *Physical Review B* **83**, 075122 (2011). URL `https://link.aps.org/doi/10.1103/PhysRevB.83.075122`.

[5] Gubernatis, J. E., Jarrell, M., Silver, R. N. & Sivia, D. S. Quantum Monte Carlo simulations and maximum entropy: Dynamics from imaginary-time data. *Physical Review B* **44**, 6011–6029 (1991). URL `https://link.aps.org/doi/10.1103/PhysRevB.44.6011`.

[6] Hancock, J. N. *et al.* Evidence for core-hole-mediated inelastic x-ray scattering from metallic $Fe_{1.087}Te$. *Physical Review B* **82**, 020513 (2010). URL `http://link.aps.org/doi/10.1103/PhysRevB.82.020513`.

[7] Zhou, K.-J. *et al.* Persistent high-energy spin excitations in iron-pnictide superconductors. *Nature Communications* **4**, 1470 (2013). URL `http://www.nature.com/ncomms/journal/v4/n2/full/ncomms2428.html`.

[8] Pelliciari, J. *et al.* Presence of magnetic excitations in SmFeAsO. *Applied Physics Letters* **109**, 122601 (2016). URL `http://scitation.aip.org/content/aip/journal/apl/109/12/10.1063/1.4962966`.

[9] Pelliciari, J. *et al.* Local and collective magnetism of $EuFe_2As_2$. *Physical Review B* **95**, 115152 (2017). URL `https://link.aps.org/doi/10.1103/PhysRevB.95.115152`.

[10] Rahn, M. C. *et al.* Paramagnon dispersion in β-FeSe observed by Fe L-edge resonant inelastic x-ray scattering. *Physical Review B* **99**, 014505 (2019). URL `https://link.aps.org/doi/10.`

1103/PhysRevB.99.014505.

[11] Garcia, F. A. *et al.* Anisotropic magnetic excitations and incipient Neel order in $Ba(Fe_{1-x}Mn_x)_2As_2$. *Physical Review B* **99**, 115118 (2019). URL https://link.aps.org/doi/10.1103/PhysRevB.99.115118.

[12] Kumar, U. *et al.* Spectroscopic signatures of next-nearest-neighbor hopping in the charge and spin dynamics of doped one-dimensional antiferromagnets. *Physical Review B* **102**, 075134 (2020). URL https://journals.aps.org/prb/abstract/10.1103/PhysRevB.102.075134

[13] Kumar, U. *et al.* Theoretical study of the spin and charge dynamics of two-leg ladders as probed by resonant inelastic x-ray scattering. *Physical Review B* **99**, 205130 (2019). URL https://journals.aps.org/prb/abstract/10.1103/PhysRevB.99.205130
